# Optimum iron-pyrophosphate electronic coupling to improve electrochemical water splitting and charge storage

**DOI:** 10.1186/s11671-023-03937-y

**Published:** 2023-12-04

**Authors:** Rishabh Srivastava, Himanshu Chaudhary, Anuj Kumar, Felipe M. de Souza, Sanjay R. Mishra, Felio Perez, Ram K. Gupta

**Affiliations:** 1 Department of Physics, Pittsburg State University, Pittsburg, KS 66762 USA; 2 National Institute for Materials Advancement, Pittsburg State University, Pittsburg, KS 66762 USA; 3 Nano-Technology Research Laboratory, Department of Chemistry, GLA University, Mathura, Uttar Pradesh 281406 India; 4 Department of Physics and Materials Science, The University of Memphis, Memphis, TN 38152 USA; 5 Integrated Microscopy Center, The University of Memphis, Memphis, TN 38152 USA; 6 Department of Chemistry, Pittsburg State University, Pittsburg, KS 66762 USA

**Keywords:** Transition metal pyrophosphate, Electrocatalysts, OER, HER, Water-splitting, Electrolyzer, Supercapacitor

## Abstract

Tuning the electronic properties of transition metals using pyrophosphate (P_2_O_7_) ligand moieties can be a promising approach to improving the electrochemical performance of water electrolyzers and supercapacitors, although such a material’s configuration is rarely exposed. Herein, we grow NiP_2_O_7_, CoP_2_O_7_, and FeP_2_O_7_ nanoparticles on conductive Ni-foam using a hydrothermal procedure. The results indicated that, among all the prepared samples, FeP_2_O_7_ exhibited outstanding oxygen evolution reaction and hydrogen evolution reaction with the least overpotential of 220 and 241 mV to draw a current density of 10 mA/cm^2^. Theoretical studies indicate that the optimal electronic coupling of the Fe site with pyrophosphate enhances the overall electronic properties of FeP_2_O_7_, thereby enhancing its electrochemical performance in water splitting. Further investigation of these materials found that NiP_2_O_7_ had the highest specific capacitance and remarkable cycle stability due to its high crystallinity as compared to FeP_2_O_7_, having a higher percentage composition of Ni on the Ni-foam, which allows more Ni to convert into its oxidation states and come back to its original oxidation state during supercapacitor testing. This work shows how to use pyrophosphate moieties to fabricate non-noble metal-based electrode materials to achieve good performance in electrocatalytic splitting water and supercapacitors.

**Supplementary Information:**

The online version contains supplementary material available at 10.1186/s11671-023-03937-y.

## Introduction

The current agenda of finding renewable energy resources to meet the global energy crisis is one of the most pressing research hotspots. In this context, the development of efficient, clean, and sustainable energy technologies, such as electrolyzers and supercapacitors, is considered the most promising approach [1–3]. However, these systems require low-cost and highly efficient electrode materials for commercialization [4–6]. To date, electrode material research has concentrated on discovering materials that can meet both low- and high-energy needs while being inexpensive, highly active in water electrocatalysis, displaying high specific energy and power density in supercapacitors, and having great cycling stability [7, 8]. Within this framework, transition metal oxides [RuO_2_, MnO_2_, Co_3_O_4_, hydroxides (Ni(OH)_2_, Co(OH)_2_), sulfides (MoS_2_, Co_9_S_8_), and phosphides (Ni_2_P)] have been tested as electrode materials in these devices [9–15]. However, these materials still have limited electrochemical performance and stability in water electrolyzers and supercapacitors, which may be due to poor alteration of transition metal electronic characteristics by corresponding oxide, sulfide, and phosphide frameworks [15–20].

Recently, it was hypothesized that the pyrophosphate framework would demonstrate exceptional electronic alteration of transition metal atoms as well as possess outstanding physical and chemical properties. For example, Mn, Co, and Ni transition metals were integrated into a pyrophosphate framework, forming Mn_2_P_2_O_7_, Co_2_P_2_O_7_, and Ni_2_P_2_O_7_. These materials have attracted substantial attention from researchers for improved water-splitting performance and energy storage capacity [21, 22]. For instance, sodium ion-doped, and amorphous Ni_2_P_2_O_7_, and porous Co_2_P_2_O_7_, having different morphologies, were constructed and found to be promising materials in energy storage applications [23]. Although pyrophosphate-based materials worked well and showed promising electrochemical performance in energy storage devices, it would be ideal to develop a simple and economical way to boost their electrochemical water oxidation performance [24].

Besides, disordered and defect-rich amorphous nanostructured materials with high mechanical and electric isotropy and low crystallinity can have interesting physico-chemical properties. Amorphous nanostructures permit the deeper dispersion of the electrolyte’s ionic species to approach the active components, benefiting from their charge storage features in supercapacitors compared to similar crystallized materials [25–28]. With the exception of the substantial amount of work that has been done on transition metal integrated pyrophosphate materials for applications involving energy storage, these materials have been inadequately investigated in electrocatalytic water splitting. In addition, theoretical research on pyrophosphate electrode materials is still limited to investigating the underlying chemistry of the influence that the framework of pyrophosphate has on transition metal atoms [29].

Therefore, in light of these considerations and to investigate the possible applications of metal-pyrophosphate-type materials in both energy conversion and storage, the authors directly grown NiP_2_O_7_, CoP_2_O_7_, and FeP_2_O_7_ nanoparticles for the first time on conductive Ni-foam using the hydrothermal approach, and the electrocatalytic and electrochemical characterizations were investigated under the prelims of surface phenomena. Since we have utilized urea during synthesis, it also reacts with Ni-foam and it is relevant to errors if we calculate mass loading on the Ni-foam. Thus, we have not proceeded with this study on the basis of mass-loading. However, we highly consider mass-loading as a crucial parameter to report for electrochemical explanation. Therefore, we already measured the mass-loading before testing. The FeP_2_O_7_ composite showed the lowest overpotential of 220 and 241 mV @10 mA/cm^2^ for the oxygen evolution reaction (OER) and hydrogen evolution reaction (HER). Further, the NiP_2_O_7_ composite was found to have the highest specific capacitance as well as the best cycle stability for all the composites. According to theoretical studies, the optimal electronic coupling of the Fe site with the pyrophosphate framework is what contributes to the enhancement of FeP_2_O_7_'s overall electronic properties and benefits to its electrochemical performances in water splitting.

## Experimental study

### Materials

Nickel foam (NF) was purchased from MTI-KJ Group, Richmond, California; nickel nitrate [Ni(NO_3_)_2_] and cobalt nitrate [Co(NO_3_)_2_] were ordered from STREM Chemicals; ferrous nitrate [Fe (NO_3_)_2_] and urea (CH_4_N_2_O) were acquired from ACROS Organics; deionized (D.I.) water was purchased from Fischer Scientific, USA; and sodium dihydrogen phosphate (NaH_2_PO_4_) was ordered from Sigma Aldrich.

### Preparation of the catalytic samples

Typically, a two-step protocol was followed for the preparation of transition metal-based pyrophosphate nanoparticles grown on the Ni-foam. In the first step of the synthesis, 3 mmol of sodium dihydrogen phosphate, 240 mg of urea, and 20 ml of de-ionized water were added with 1 mmol of nickel nitrate, cobalt nitrate, and ferrous nitrate precursors in three different beakers, respectively, and the resulting solutions were sonicated for 5 min at 25 °C to ensure homogeneity of composition. In the second step, all the prepared solutions and the Ni-foams with an area of 2 × 2 cm^2^ were transferred into 50 ml Teflon autoclaves and treated at 180 °C for 12 h. After cooling down, the obtained Ni foams supported by NiP_2_O_7_, CoP_2_O_7_, and FeP2O7 arrays were washed with DI water many times before being placed in an oven for 12 h to dry.

### Physical characterization

The synthesised materials underwent characterization using an X-ray diffractometer (Shimadzu XRD-6000 with Cu-Kα source and 1.54 Å wavelength) within the angular range of two theta degrees from 10 to 80. The investigation involved the examination of morphology, elemental mapping, and energy dispersive X-ray analysis (EDAX, Oxford) utilising field emission scanning electron microscopy (FESEM) (SU 5000 – Hitachi). The X-ray photoelectron spectra (XPS) were collected at Kratos Axis Ultra-DLD spectrometer (employing Al Kα,x non-monochromatic radiation of approx. 1 mm). Elemental quantitative analysis was performed by calculating the area under the elemental peaks by fitting a curve to the data.

### Electrochemical characterization

The electrochemical experiments were conducted on a Versa STAT 4-500 electrochemical workstation using a three-electrode device. Due to the fact that the material was not grown directly on Ni-foam and mass-loading of material on Ni-foam was not considered for this study, the potentiodynamic and electrochemical supercapacitor investigation was done on the basis of surface area analysis. However, mass loading is an important parameter during electrochemical investigations therefore, we have measured mass-loading of the material on the Ni-foam. The mass-loading of NiP_2_O_7_, CoP_2_O_7_, and FeP_2_O_7_ are 26.3, 31.8, and 21.9 mg/cm^2^, respectively [30]. To analyze the catalytic activity, 1 M KOH solution was used as an electrolyte, with a saturated calomel electrode, graphite rod, and a prepared electrode serving as a reference electrode, counter electrode, and working electrode, respectively. The electrochemical characterization of HER and OER was performed using linear sweep voltammetry; all the polarization curves were plotted after the iR correction. The degree of iR compensation for NiP_2_O_7_, CoP_2_O_7_ and FeP_2_O_7_ samples was found to be 15%, 13%, and 12%, respectively [31–33]. Tafel slope, turnover frequency (TOF), electrochemical active surface area (ECSA), roughness factor (RF), electrochemical impedance spectroscopy (EIS), and chronoamperometry (CA) were also performed to evaluate the performance of the prepared samples. Additionally, for the energy storage characteristics, 3 M KOH solution was used as an electrolyte with a mercury/mercury oxide (Hg/HgO) electrode, platinum wire, and a prepared electrode serving as a reference electrode, counter electrode, and working electrode. The supercapacitor properties were investigated by accompanying cyclic voltammetry (CV), galvanostatic charge–discharge (GCD), and durability tests over 5000 cycles using a three-electrode system. The turnover frequency (TOF) value was calculated using the formula [34, 35];$TOF=J{N}_{A} /nF\tau$, where, J, NA, n, F, and τ stands for the current density (A/cm^2^), Avogadro number (6.022 × 1023 mol^−1^), transferred electrons during product formation (for O_2_, it is 4, and H_2_, it is 2), Faraday constant (96,485 C), and surface concentration of active sites, respectively.

### Theoretical studies

To perform the theoretical calculations, the primitive unit cell with lattice parameters of a = 10.14 Å and b = 12.22 Å, having 20 Å vacuum space, was constructed for each catalytic material. The Quantum Espresso calculation software was used to do theoretical computations on the constructed models by employing the generalized-gradient approximation (GGA) theory in association with the Perdew–Burke–Ernzerhof (PBE) functional and the double numerical (+) polarisation functional basis set. To calculate the density of states (DOS) for the constructed models, the convergence limits for force (0.05 eV) and energy (250 eV) were set under the plane-wave expansion of the electronic wave function (10^−5^ eV). After thoroughly optimizing the constructed models, the adsorption energies for OER and HER adducts were calculated using the following Eq. 1.

1 $$\Delta {\text{E}}_{{{\text{ads}}.}} = {\text{E}}_{{({\text{catalyst}})}} + {\text{E}}_{{({\text{reactant}})}} - {\text{E}}_{{({\text{catalyst}} - {\text{reactant}})}}$$

where E_(catalyst-reactant)_, E_(catalyst)_, and E_(reactant)_ are the energies of catalyst-reactant adduct, catalyst, and reactant, respectively.

## Results and discussions

### Synthesis, and characterization of the prepared samples

**Fig. 1:**
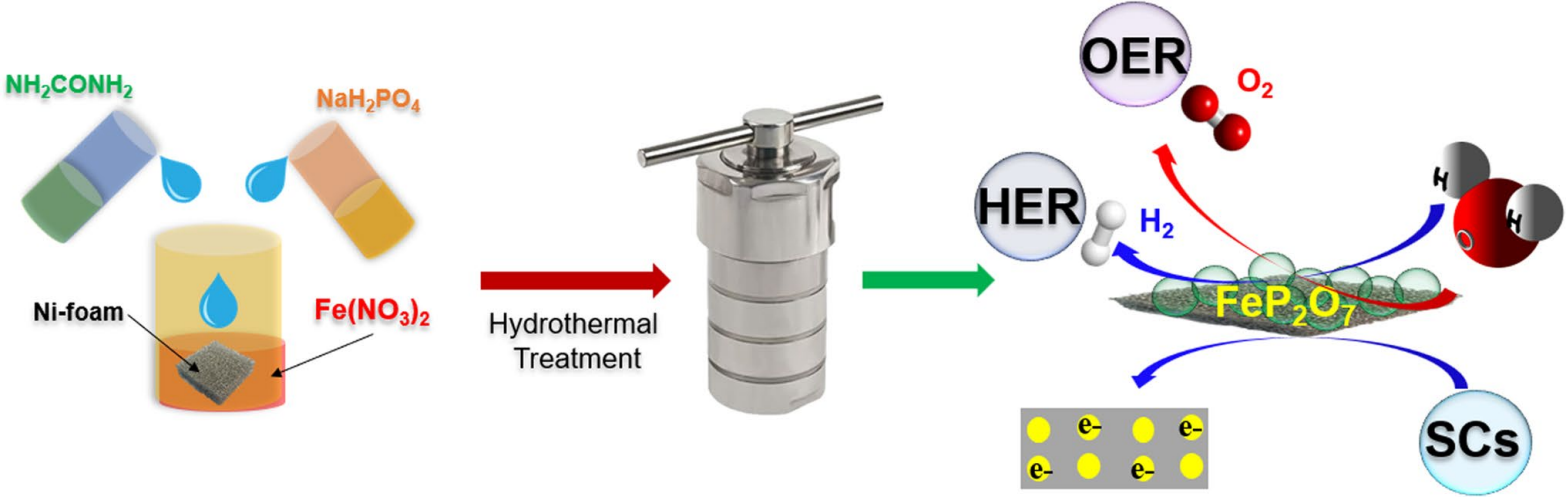
A schematic representation of a two-step preparation of NiP_2_O_7_, CoP_2_O_7_, and FeP_2_O_7_ in hydrothermal

In this study, to investigate the electrochemical performance of transition metal-based pyrophosphate-type materials towards electrocatalytic water oxidation and supercapacitors, the FeP_2_O_7_, CoP_2_O_7_, and NiP_2_O_7_ nanoparticles were grown on conductive Ni-foam, following a two-step strategy, as shown in Fig. 1. First of all, all the prepared composite materials were characterized using multiple analytical techniques and then investigated for the electrochemical performances using electrochemical techniques.

**Fig. 2:**
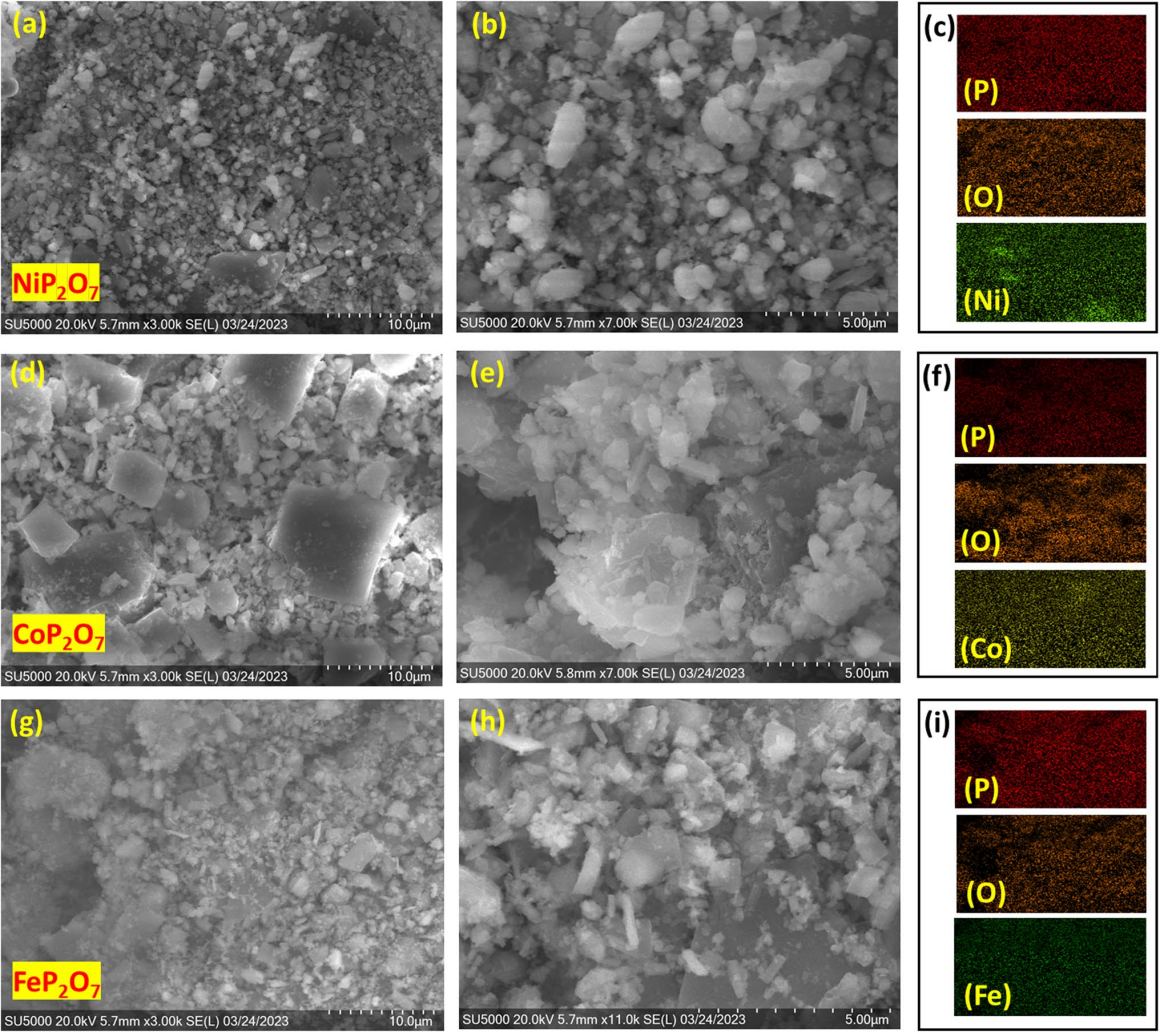
SEM images of powdered **a**, **b** NiP_2_O_7_, **d**, **e** CoP_2_O_7_, and **g**, **h** FeP_2_O_7_. Elemental mapping at 10 μm of **c** P, O, and Ni for NiP_2_O_7_, **f** P, O, and Co for CoP_2_O_7_, and **i** P, O, and Fe for FeP_2_O_7_

The morphology of the prepared composites, FeP_2_O_7_, CoP_2_O_7_, and NiP_2_O_7_, were characterized by the SEM technique. The SEM pictures (Fig. 2) of these prepared composites (powdered form) showed almost similar morphology, having a mixture of microplate and agglomerated nanoparticles with non-homogeneous sizes of 15–1 µm of FeP_2_O_7_, CoP_2_O_7_, and NiP_2_O_7_ NPs, which were distributed over the Ni-foam. Further, SEM–EDX mapping was carried out at 10 μm to analyze the composition of the prepared samples. The results indicated that images taken under the NiP_2_O_7_, CoP_2_O_7_, and FeP_2_O_7_ samples have corresponding elements, as shown in the right inset figure of Fig. 2 and the respective EDX of the as-prepared samples were illustrated in Fig. S1. Moreover, to gain a further understanding on the microstructure of the as prepared materials, the SEM of NiP_2_O_7_, CoP_2_O_7_, and FeP_2_O_7_ grown on Ni-foam were taken at different magnifications as well as accounted elemental mapping and EDX analysis for the same in Figs. S2, S3, and S4, respectively. After observing SEM taken before testing reveal the similar microplate-like structure for all the three prepared samples.

**Fig. 3:**
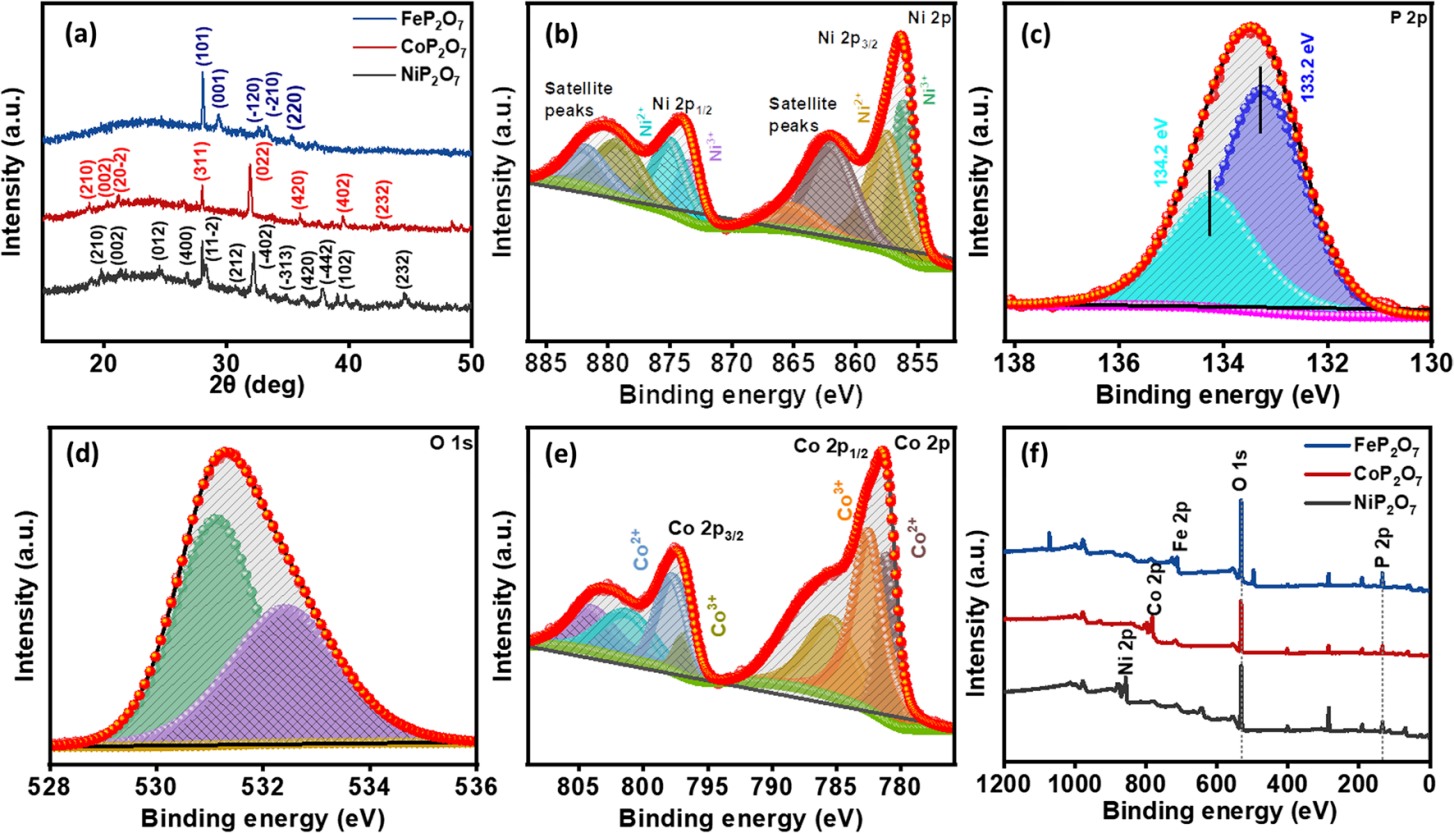
**a** XRD spectra of NiP_2_O_7_, CoP_2_O_7_, and FeP_2_O_7_, High-resolution XPS spectra of the NiP_2_O_7_, **b** Ni 2*p*, **c** P 2*p*, **d** O 1*s*, **e** Co 2*p* XPS spectra of CoP_2_O_7_, and **f** XPS survey spectra of NiP_2_O_7_, CoP_2_O_7_, and FeP_2_O_7_

Further, to characterize the sample purity, crystallinity, and crystal structure of the prepared NiP_2_O_7_, CoP_2_O_7_, and FeP_2_O_7_ samples, XRD spectra were recorded. The XRD pattern (Fig. 3a) of the prepared catalytic samples showed that NiP_2_O_7_, CoP_2_O_7_, and FeP_2_O_7_ samples were perfectly matched with JCPDS#74-1604 [36–39], JCPDS#34-0378 [40–44], and JCPDS#72-1516 [45–48], respectively, confirming their precise structure, as shown in the theoretical section. Subsequently, X-Ray Photoelectron Spectroscopy was employed to investigate the elemental chemical states and results reveal the main elements such as Ni, Co, Fe, P, and O in NiP_2_O_7_, CoP_2_O_7_, and FeP_2_O_7_. Furthermore, the high-resolution XPS of Ni 2*p*, P 2*p*, and O 1*s* of NiP_2_O_7_ are displayed in Fig. 3b–d, respectively. In Fig. 3b, the two peaks are obtained after Gaussian fitting of the Ni 2p spectrum located at 875.4 and 857.5 eV corresponding to Ni 2*p*_1/2_ and Ni 2*p*_3/2_, respectively. Moreover, the satellite peaks at 880.5 and 862.1 eV are attributed to Ni (II) [49]. In addition, the peaks located at 133.2 and 134.2 eV in Fig. 3c, correspond to the significant P 2*p*_3/2_ designated to P (V), and P 2*p*_1/2_, respectively, and ΔE between the two characteristic peaks is 1 eV. The P 2*p* spectrum further confirmed the presence of (P_2_O_7_)^4−^ at binding energy 133.2 eV. The wider O 1s spectrum in Fig. 3d was deconvoluted into two Gaussian distributions corresponding to two prominent species, chemisorbed hydroxyl (OH) at 533 eV and lattice oxygen at 531.8 eV. Figure 3e shows the high-resolution XPS spectrum of Co, and Figs. S5 and S6 delineate P and O XPS spectra for CoP_2_O_7_, respectively. The Co 2*p* compromised of the Co 2*p*_3/2_ and Co 2*p*_1/2_ peaks located at 781.5 and 797.5 eV, furthermore the two shake-up satellite peaks located at 786.1 and 802.7 eV, clearly confirms the presence of Co (II) in the obtained material [50]. The peaks designated at specific binding energy correspond to the characteristic P 2*p*_3/2_ peaks of P (V) [50]. The peaks in Fig. S6 illustrate the main peaks of O 1*s*, confirming the presence of O (II). Simultaneously, the high-resolution XPS spectra of Fe 2*p*, P 2*p*, and O 1*s* in Figs. S7–S9 for FeP_2_O_7_ were obtained, respectively. The XPS spectra of Fe 2*p* contain Fe (III) at 711.4 eV, and Fe (II) at 709.7 and 714.2 eV. Thus, the ratio between Fe (III) and Fe (II) components in the XPS is approximately close to 1:2 [51]. Moreover, the XPS P 2*p* and O 1*s* consistently suggest a partial negative charge of pyrophosphate ion and due to electron transfer from Fe and P_2_O_7_ ions [51, 52]. Further, the survey spectrum evident the presence of Ni, Co, Fe, P, and O in the as-synthesized samples, displayed in Fig. 3f, which comprehensively suggests the formation of NiP_2_O_7_, CoP_2_O_7_, and FeP_2_O_7_, combining the results from XRD, further confirmed the formation of the samples with XPS observations.

### Electrochemical performance

#### Hydrogen evolution reaction

**Fig. 4:**
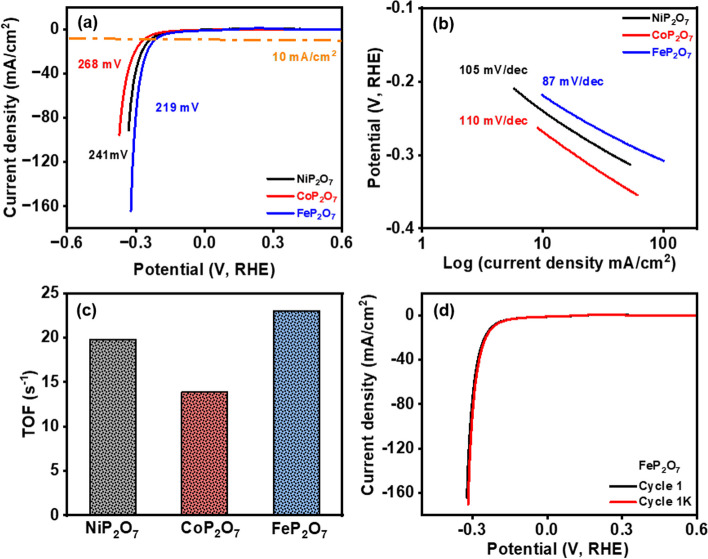
**a** Overpotential at 10 mA/cm^2^ for NiP_2_O_7_, CoP_2_O_7_, and FeP_2_O_7_, **b** Tafel slopes, **c** TOF at 100 mV of HER performance for all nanomaterial electrodes, and **d** LSV curves after and before 1000 cycles of cyclic voltammetry for FeP_2_O_7_ sample. All the LSV curves were recorded at 2 mV/s scan rate in 1 M KOH electrolyte

Electrocatalytic HER activity of the prepared NiP_2_O_7_, CoP_2_O_7_, and FeP_2_O_7_ samples was assessed in 1 M KOH electrolyte using the LSV technique. Figure 4a shows the typical HER polarization curves for FeP_2_O_7_, CoP_2_O_7_, and NiP_2_O_7_ samples, demonstrating 219 mV, 268 mV, and 241 mV, respectively, at 10 mA/cm^2^ current density. FeP_2_O_7_ exhibited superior HER activity as compared to CoP_2_O_7_ and NiP_2_O_7_ samples (Fig. S10). Further, to observe the HER kinetics, the Tafel plots for these samples were drawn against their respective LSV data. Figure 4b confirms that the rate-determining step (RDS) for these samples followed the Volmer-Heyrovsky reaction route (H_2_O + e^−^ → H_ads_ + OH^−^). From Fig. 4b**,** it is clear that the FeP_2_O_7_ sample showed the lowest Tafel slop value of 87 mV/dec, as compared to CoP_2_O_7_ (110 mV/dec), and NiP_2_O_7_ (105 mV/dec) samples, suggesting its fast HER kinetics as well as remarkable HER performance. Further, to support the RDS, the TOF values for these catalytic samples were calculated (Fig. 4c), and the results suggested the acquisition of a TOF value of 23.02 s^−1^ by FeP_2_O_7_, which is much higher compared to CoP_2_O_7_ and NiP_2_O_7_ samples. Therefore, the low overpotential of the FeP_2_O_7_ sample corresponds to a higher TOF value, indicating its outstanding HER performance along with efficient rate transfer as compared to CoP_2_O_7_ and NiP_2_O_7_ samples. Further, to check the practical applicability of the FeP_2_O_7_ sample, a durability test was also performed using linear sweep voltammetry. The HER LSV curve for the FeP_2_O_7_ sample after 1000 CV cycles displayed negligible fluctuation in its onset potential as well as current density as compared to the first CV cycle, demonstrating its significant durability during the HER process (Fig. 4d). Similar observation was found for NiP_2_O_7_ and CoP_2_O_7_ (Figs. S11, S12).

**Fig. 5:**
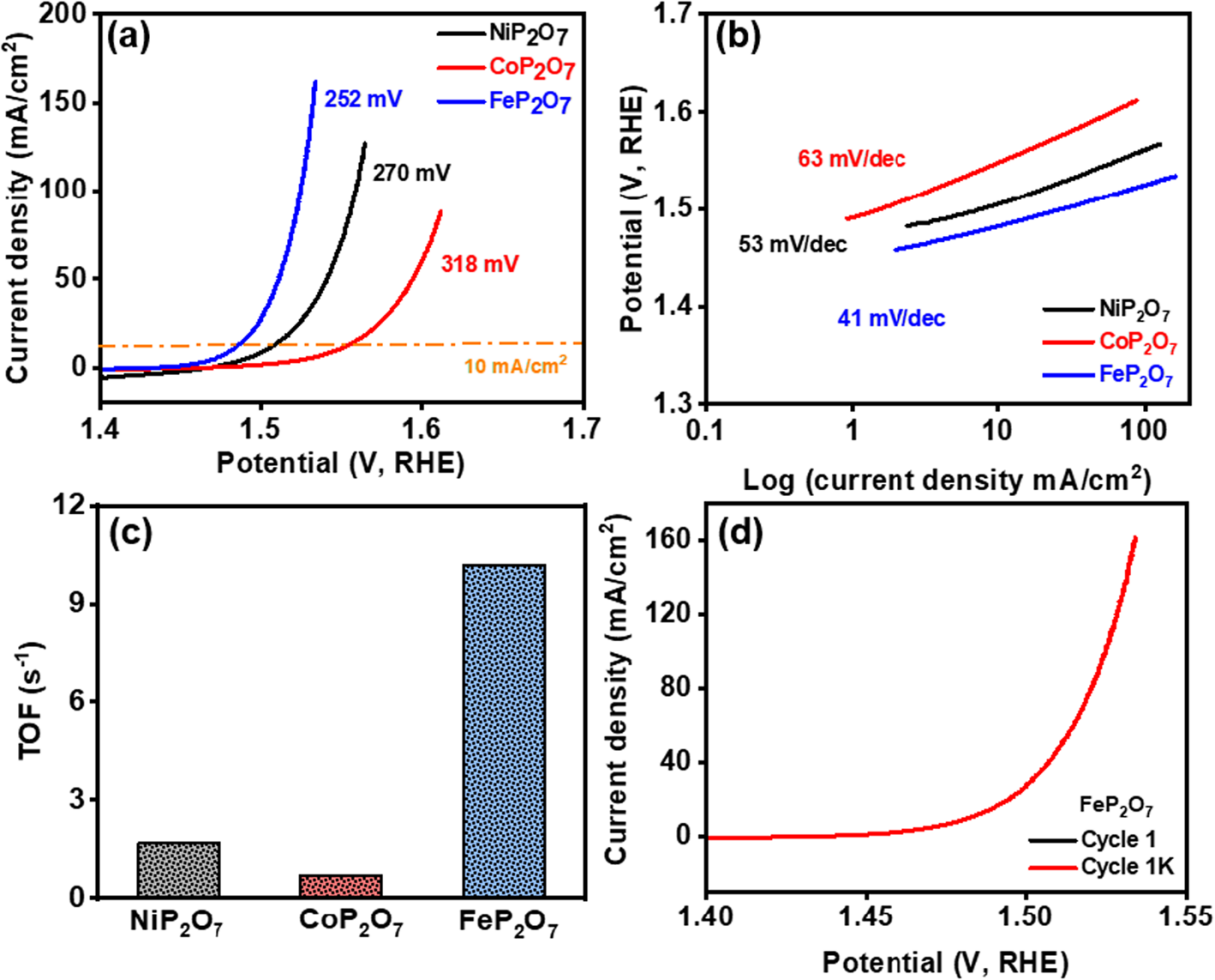
**a** Overpotential at 10 mA/cm^2^ for NiP_2_O_7_, CoP_2_O_7_, and FeP_2_O_7_. All the LSV curves were recorded at 2 mV/s scan rate in 1 M KOH electrolyte. **b** Tafel slopes, **c** TOF at 350 mV of HER performance for all nanomaterial electrodes, and **d** LSV curves after and before 1000 cycles of cyclic voltammetry for FeP_2_O_7_ sample

Further, the prepared samples were evaluated for their OER performance using the LSV technique in 1 M KOH media. The iR corrected LSV plots (Fig. 5a) reveal that to attain the 10 mA/cm^2^ current density, FeP_2_O_7_ displayed a 252 mV onset overpotential, which is significantly lower than CoP_2_O_7_ (318 mV) and NiP_2_O_7_ (270 mV) samples (Fig. S13). In addition, to derive the value of Tafel slopes (Fig. 5b), the Butler–Volmer equation was utilized. A low Tafel value (41 mV/dec) for FeP_2_O_7_ is an indication of improved, faster OER kinetics than CoP_2_O_7_ and NiP_2_O_7_ samples. The TOF value for these samples was also calculated to analyze their intrinsic kinetic behaviour [53]. The TOF value for FeP_2_O_7_, CoP_2_O_7_, and NiP_2_O_7_ samples was found to be 10.17, 0.69, and 1.71 s^−1^, respectively, indicating the facile OER with the FeP_2_O_7_ sample (Fig. 5c). In addition, the OER LSV curve for the FeP_2_O_7_ sample recorded before and after 1000 CV cycles displayed negligible fluctuation in its onset potential as well as current density, demonstrating its significant durability during the OER process (Fig. 5d). Similar observation was found for NiP_2_O_7_ and CoP_2_O_7_ (Figs. S14, S15).

**Fig. 6:**
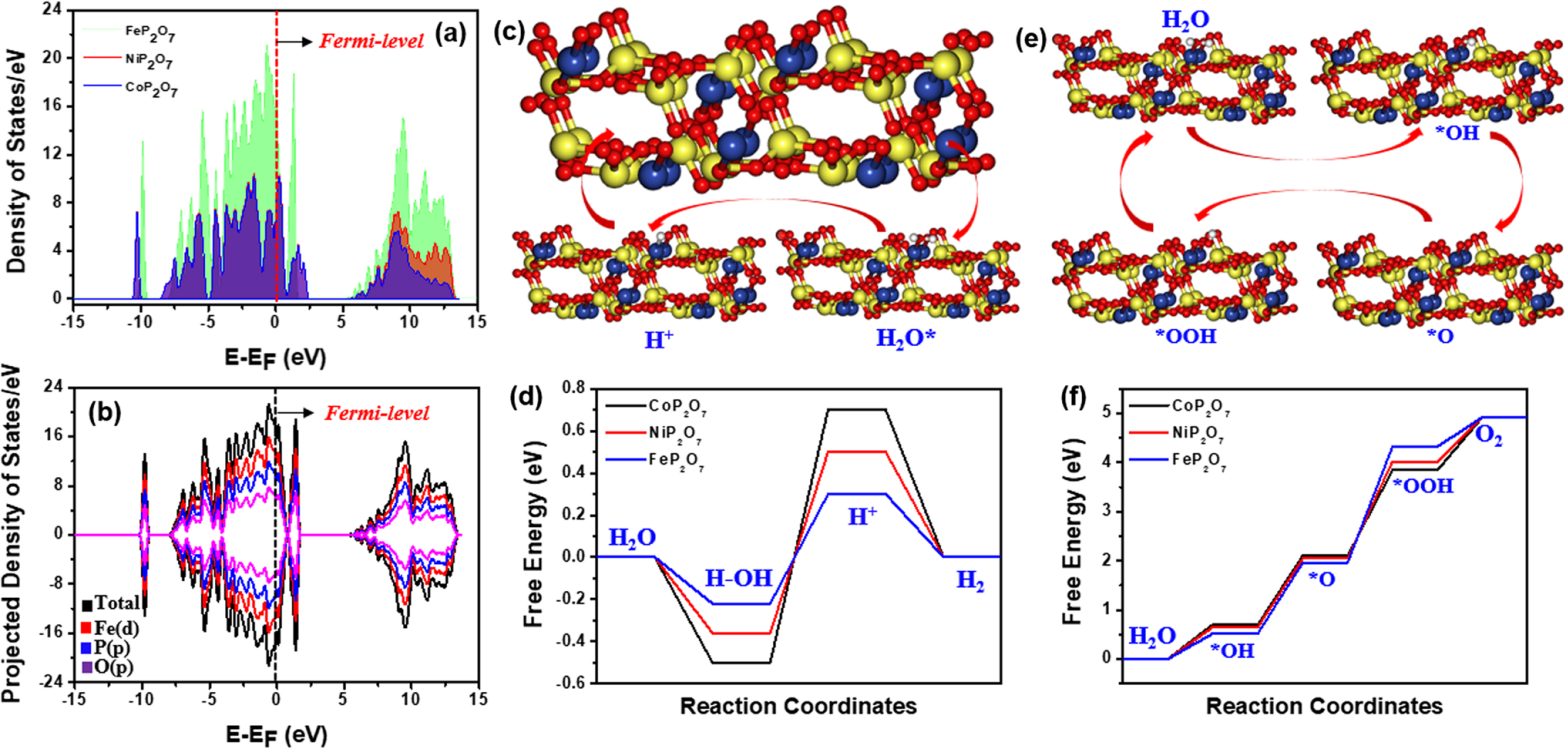
**a** Density of states (DOS) of NiP_2_O_7_, CoP_2_O_7_, and FeP_2_O_7_ samples, and **b** Projected Density of states (PDOS) of FeP_2_O_7_ sample. **c** HER mechanism, **d** HER Free energy diagram, **e** OER mechanism, and **f** OER Free energy diagram, for FeP_2_O_7_ sample

Further, to confirm the catalytic OER and HER mechanisms for the prepared NiP_2_O_7_, CoP_2_O_7_, and FeP_2_O_7_ samples and to find the reasons for the improved catalytic performance of FeP_2_O_7_ sample as compared to CoP_2_O_7_, and NiP_2_O_7_ samples, theoretical calculations was performed using DFT. First, of all, we optimized the constructed slabs for these catalytic models, as shown in Fig. 6a. The optimized energy indicated the higher stability of Fe-atom with pyrophosphate. After that, we calculated the density of states for these samples (Fig. 6b), and found the higher high density of state (DOS) and its distribution over the fermi level for the FeP_2_O_7_ sample, as compared to CoP_2_O_7_, and NiP_2_O_7_ samples**.** These results suggested that the pyrophosphate framework significantly improved the electronic properties of Fe-atom as compared to Co and Ni atoms, benefiting to catalytic performance of the FeP_2_O_7_ sample towards HER and OER, as supported by experimental investigations. Further, to confirm the HER mechanism on the prepared samples, the transition metal atom was considered as the active site (*). For HER, H_2_O adsorption (H_2_O* state), H-adsorption (H* state)**,** and H_2_ disposal (Ni-1/2H_2_ state) on the active site were optimized (Fig. 6c). The computed corresponding free energy curves for these samples clearly indicated the less energy barrier for HER (the values for activity descriptors, ΔG_H2O*_ and ΔG_H*_, were very close to zero) on the FeP_2_O_7_ sample, which might be due to its improved electronic properties, suggesting its efficient HER activity as compared to CoP_2_O_7_, and NiP_2_O_7_ samples (Fig. 6d). In a similar way, all the intermediates of OER were optimized on the corresponding transition metal site of NiP_2_O_7_, CoP_2_O_7_, and FeP_2_O_7_ samples (Fig. 6e). The transformation of *O into *OOH can be considered as the potential determining step. Moreover, the free energy diagram suggested that the OER process followed the lower energy barrier with the FeP_2_O_7_ sample as compared to CoP_2_O_7_, and NiP_2_O_7_ samples, indicating its superior OER performance (Fig. 6f).

**Fig. 7:**
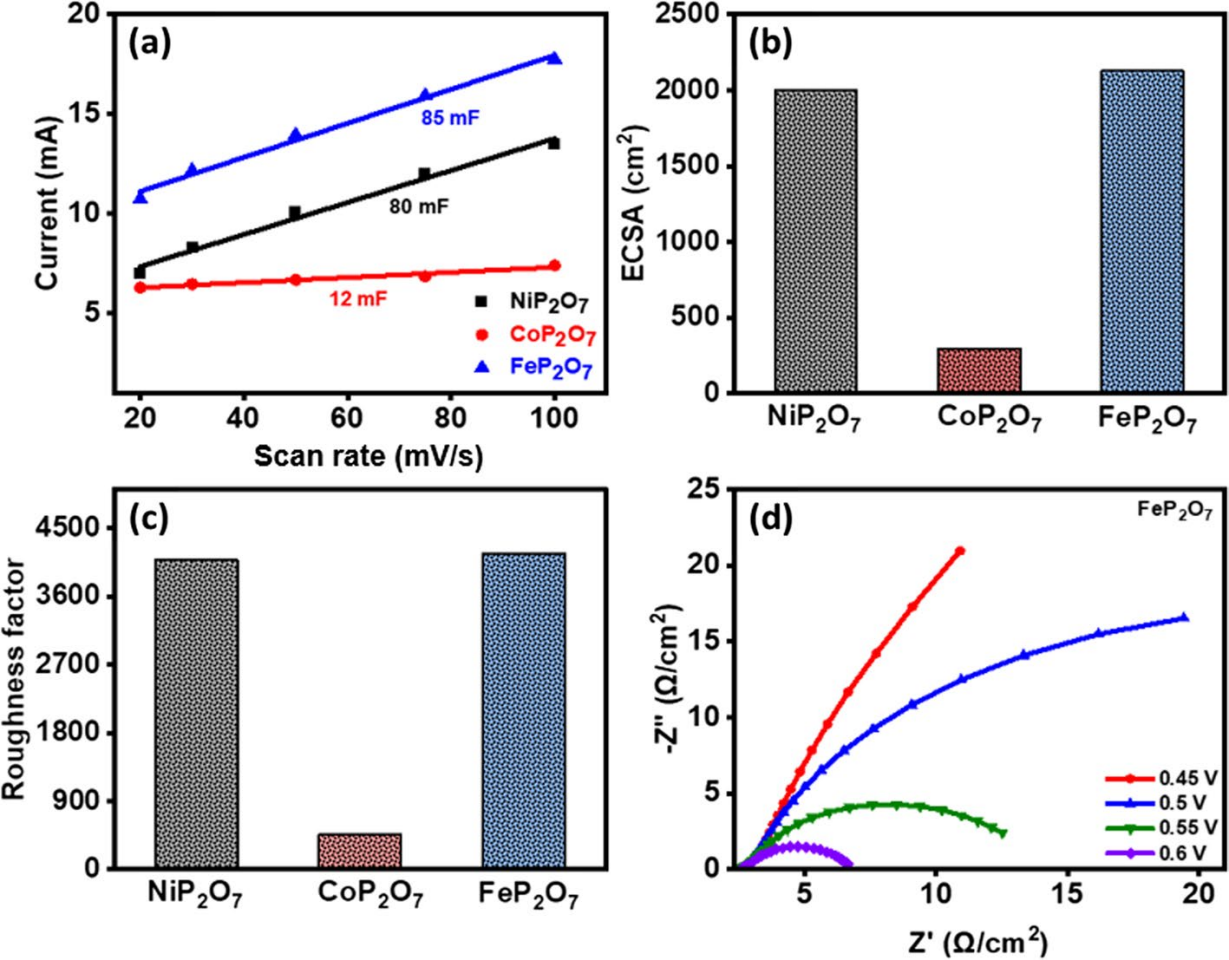
**a** Double layer capacitance of NiP_2_O_7_, CoP_2_O_7_, and FeP_2_O_7_ samples, **b** Electrochemical surface area. **c** Roughness factor, **d** Nyquist plot of FeP_2_O_7_ sample obtained from electrochemical impedance spectroscopy

**Fig. 8:**
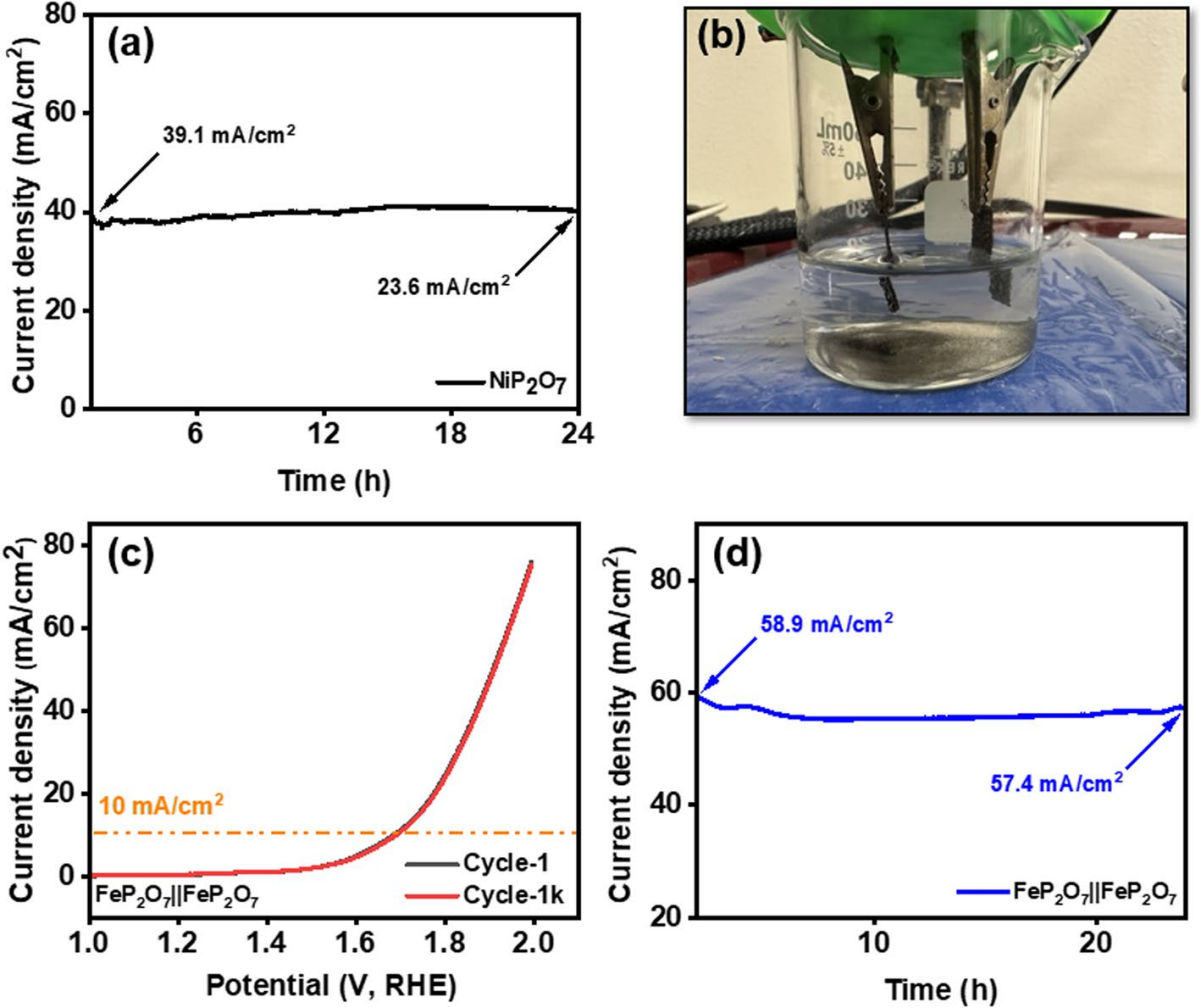
**a** Chronoamperometry curve of NiP_2_O_7_, **b** working of the electrolyzer FeP_2_O_7_||FeP_2_O_7_, **c** LSV curve for electrolyzer and polarization stability over 1000 cycles, and **d** chronoamperometry test for electrolyzer over 24 h

Further, the ECSA was calculated (using the equation; $ECSA= {C}_{dl}/{C}_{s}$, where Cs = 0.04 mF/cm^2^ in 1 M KOH [35] to correlate the improved catalytic performance of FeP_2_O_7_ sample. It is a thorough comprehension of the mechanism behind the nature of electrocatalysts by developing CV in the region that does not include oxidation or reduction [54]. In order to conduct an analysis of the ECSA, the current densities and related scan rates were plotted, as displayed in Fig. 7a. Also, the value of ECSA is proportional to the value of the electrochemical double-layer capacitance (C_dl_). Therefore, the higher the value of C_dl_, the greater the ECSA (Fig. 7b), and the more effective the catalytic activity. The C_dl_ values for the NiP_2_O_7_, CoP_2_O_7_, and FeP_2_O_7_ samples are 80, 12, and 85 mV, respectively. FeP_2_O_7_ samples have an ECSA of 2125 cm^2^, which is remarkably higher than CoP_2_O_7_ and NiP_2_O_7_ samples, indicating more exposure of their active sites towards the catalytic reactions and thereby improving their catalytic performance in OER and HER. In that sense, the roughness factor was calculated to observe the material performance toward producing gas bubbles efficacy as shown in Fig. 7c. Thus, FeP_2_O_7_ delineates the highest roughness factor than other samples, indicating the highest ECSA value corresponds to the greater number of active sites for catalytic reaction to produce more gas bubbles supported by the roughness factor. Furthermore, to study the motion of the ions under the electrochemical atmosphere we carried out an electrochemical impedance spectroscopy test to analyze the charge transfer resistance (R_ct_). Thus, the Nyquist plot of all the samples was plotted in Fig. 7d, Figs. S16 and S17 of FeP_2_O_7_, NiP_2_O_7_, and CoP_2_O_7_, respectively at different potentials of 0.45, 0.5, 0.55, and 0.6 V. Moreover, a fitted Randles circuit is demonstrated in Fig. S18 to exaggerate the attribute of Nyquist plot [55–59] and After analyzing the Nyquist plot at various potential, it was noteworthy found that FeP_2_O_7_ showed least charge transfer resistance among all the samples and over different potentials, tabulated in Table S1 in the supplementary information. Furthermore, the longevity stability of the as-prepared samples was examined by using Chronoamperometry testing over 24 h. Figure 8a and Fig. S19a, b displayed a chronoamperometry curve of NiP_2_O_7_, and CoP_2_O_7_, and FeP_2_O_7_, respectively, at 0.65 V potential. The current density drop was fluctuated from 39.1, 47.2, and 114.6 to 23.6, 39.1, and 109.2 mA/cm^2^ of NiP_2_O_7_, CoP_2_O_7_, and FeP_2_O_7_, respectively without any extreme deterioration. It was found that FeP_2_O_7_ has a lowest current density drop of about 5.4 mA/cm^2^ than other materials. However, a variation was caused after 12 h due to release of bubble during water-splitting.

Since the FeP_2_O_7_ sample showed excellent bifunctional OER and HER performance, an electrocatalytic water-splitting device was built with the FeP_2_O_7_ sample serving as both anode and cathode (Fig. 8b) to test its suitability for practical application in a water electrolyzer. The results show that a sample of FeP_2_O_7_ can faithfully adhere to either a 2- or 4-electron process over an extended period of time. The FeP_2_O_7_||FeP_2_O_7_ on NF showed a low cell potential of just 1.69 V at a current density of 10 mA/cm^2^ (Fig. 8c), while still creating a huge bubble with a high endurance, as seen by the overlap between the 1st and 1000th cycles of polarization curves (Fig. 8c). Then, long term durability test was performed for 24 h (Fig. 8d) and it was observed that the electrolyzer attained 58.9 mA/cm^2^ of current density and after 24 h it goes down to 57.4 mA/cm^2^, indicating highly stable device material for splitting water into H_2_ and O_2_. In order to analyze the durability of the FeP_2_O_7_ material electrode towards overall water-splitting process, the XRD and SEM images were taken after overall water-splitting stability testing as enumerated in Fig. S20, indicating no structural changes depicted from XRD and no significant change in the morphology was observed through SEM. Due to its robust nature, it can be summarized that there is no obvious chemical changes after overall water-splitting and can be a best candidate towards HER and OER activity.

#### Supercapacitor study

After microscopic and phase investigations of the materials, the detailed performance of the prepared metal-based pyrophosphate electrodes toward the electrochemical supercapacitor using cyclic voltammograms (CV), galvanostatic charge–discharge (GCD), electrochemical impedance spectroscopy (EIS), and stability. Polyphosphates possess excellent chemical stability, indicating better for the long-life challenge of cycling ability. On the other hand, the electrical harvesting, kinetics of charges, and holding capacity over a long time have been challenging problems for pyrophosphate materials. Herein, we have developed the NiP_2_O_7_, CoP_2_O_7_, and FeP_2_O_7_ grown on Ni-foam to increase the overall conductivity of the electrode. Moreover, the synthesized sample was treated at an optimum temperature during hydrothermal which helped to grow metal-pyrophosphate without shrinking of material and indicated a strong bond between the material and the substrates, leading to highly enhanced stability in electrochemical reactions. For instance, Wang et al. [60] designed marigold flower-like Mn_2_P_2_O_7_ material and further applied it to a Li-ion battery. However, the obtained material was irreversible at first due to charges consumed to reduce Mn^2+^ ions to the metallic state, and owing to the formation of electrolyte interphase.

**Fig. 9:**
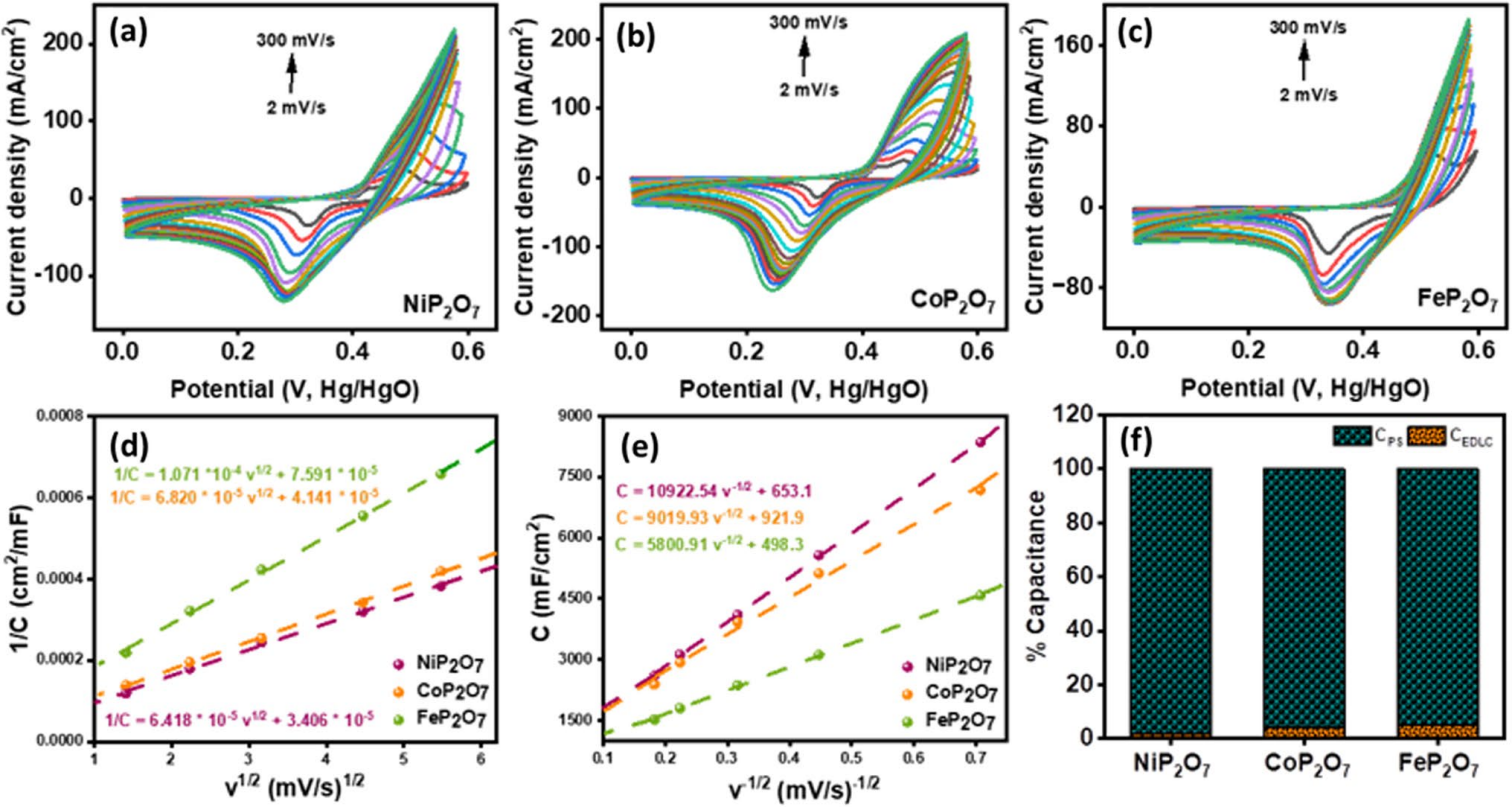
**a**–**c** CV voltammogram for the NiP_2_O_7_, CoP_2_O_7_, and FeP_2_O_7_, at different scan rates ranging from 2 to 300 mV/s, respectively, Trasatti plot of **d** 1/C versus V^1/2^ of the NiP_2_O_7_, CoP_2_O_7_, and FeP_2_O_7_, and **e** C versus v^−1/2^ of NiP_2_O_7_, CoP_2_O_7_, and FeP_2_O_7_, **f** percentage of capacitance contribution evaluated for the electrodes based on trasatti analysis

Later on, Senthilkumar et al. [61] fabricated a highly porous carbon-based Ni_2_P_2_O_7_ electrode for supercapacitor application. To promote high conductivity and stability, electrode material possesses a high-purity phase, morphology, sufficient working potential, and reversibility. In those terms, they successfully acquired grain-like nanoparticles which resembled the monoclinic phase of Ni_2_P_2_O_7_ which further enhanced the electrochemical process. Following to that, Hou et al. [62] designed promising 1D Co_2_P_2_O_7_ nanorods without any templates and high-temperature calcination as a pseudocapacitive material for energy storage. The obtained material contains CoO_6_ coordination octahedron phase with P_2_O_7_ groups as an electroactive component for electrochemical activity. In addition, its magnetic and microwave absorption properties also fascinated that material for highly stable and reversible supercapacitor devices. Considering all those parameters, the developed NiP_2_O_7_, CoP_2_O_7_, and FeP_2_O_7_ was projected towards preliminary CV testing in 3 M KOH electrolyte at various scan rate from 2 to 300 mV/s in a potential window of 0–0.6 V and the outcomes are illustrated in Fig. 9a–c. CV plot demonstrated pseudocapacitive behavior of the materials and one oxidation and reduction peak appeared in NiP_2_O_7_ and FeP_2_O_7_ at lower scan rate unless CoP_2_O_7_, having two oxidation peaks where first peak around 0.3–0.4 V can be ascribed to the oxidation of Co to Co^2+^ and peak about 0.45–0.55 occurred due to the Co^2+^ to Co^3+^; all the samples showed stable redox profile the profound reversibility due to symmetric nature of faradic reaction. Therefore, the proposed redox mechanism for NiP_2_O_7_, CoP_2_O_7_, and FeP_2_O_7_ with basic electrolyte is described in the Eq. (2–4), respectively.

2 $${\text{NiP}}_{{2}} {\text{O}}_{{7}} + {\text{OH}}^{ - } \leftrightarrow {\text{NiP}}_{{2}} {\text{O}}_{{7}} \left( {{\text{OH}}^{ - } } \right)_{{2}} + {\text{2e}}^{ - }$$

3 $${\text{CoP}}_{{2}} {\text{O}}_{{7}} + {\text{OH}}^{ - } \leftrightarrow {\text{CoP}}_{{2}} {\text{O}}_{{7}} \left( {{\text{OH}}^{ - } } \right)_{{2}} + {\text{2e}}^{ - }$$

4 $${\text{FeP}}_{{2}} {\text{O}}_{{7}} + {\text{OH}}^{ - } \leftrightarrow {\text{FeP}}_{{2}} {\text{O}}_{{7}} \left( {{\text{OH}}^{ - } } \right)_{{2}} + {\text{2e}}^{ - }$$

The electrode material delineated quasi-reversible reaction which is because of multiple oxidation states of the Ni, Co and Fe, here Ni^2+^/Co^2+^/Fe^2+^ transformed to the Ni^3+^/Co^3+^/Fe^3+^ during redox process. From the reaction Eq. (2–4), inferred that the pair of redox reaction can be observed at a specified potential range, indicating reversible reaction. Analogous to the work done by Wang et al. and group, where they acknowledged that due to lower Pauling electronegativity of P (2.19) enables much higher conductivity. Therefore, the electronegativity difference of P_2_O_7_ anion facilitates Ni, Co and Fe and without any further distortion of the CV plots, resembling best reversibility, enhanced mass transportation, capacitive property and rate of charge transfer. Furthermore, the observed distinctive peaks at lower rate corresponds to the pseudocapacitive performance. However, while increasing the scan rate the anodic peak shifted towards right and cathodic peak to the lower potential due to limited interaction of OH^−^ ions. Thereby, we employed a Trasatti method in order to identify the charge stored by the material of the electrodes, either a pseudocapacitive which refers to reversible redox phenomenon, showing the intercalation and electro absorption of the ions or electrochemical double layer capacitance (EDLC) formed at the electrode/electrolyte interface. The Eq. (5–7) are involved to study the mechanism behind the CV curves.

5 $${1}/{\text{C}} = {\text{k}}_{{1}} {\text{v}}^{{{1}/{2}}} + {1}/{\text{C}}_{{\text{T}}}$$

6 $${\text{C}} = {\text{k}}_{{2}} {\text{v}}^{{ - {1}/{2}}} + {\text{C}}_{{{\text{EDLC}}}}$$

7 $${\text{C}}_{{{\text{PS}}}} = {\text{C}}_{{\text{T}}} {-}{\text{C}}_{{{\text{EDLC}}}}$$

where C signifies areal capacitance, C_T_ is total capacitance, C_EDLC_ symbolizes electrical double layer capacitance, and C_PS_ infers pseudo capacitance contribution. A plot of 1/C in function to v^1/2^ as illustrated in Fig. 9d and a plot of C in function to v^−1/2^ as shown in Fig. 9e has been analyzed to deduce the EDLC and pseudocapacitance contribution of the NiP_2_O_7_, CoP_2_O_7_, and FeP_2_O_7_ electrodes. The overall C_EDLC_ and C_PS_ contribution was illustrated in Fig. 9f based on Trasatti method, indicating NiP_2_O_7_ offered high pseudocapacitive behavior of 98% with a least EDLC influence of 2%. However, CoP_2_O_7_ and FeP_2_O_7_ showed pseudo mechanism by 96% and 95% and EDLC of 4% and 5%, respectively.

Overall, the NiP_2_O_7_ represented pseudocapacitive dominated material. Furthermore, to corroborate the kinetic mechanism of the as synthesized materials in terms of diffusion and capacitive nature by indepth observation of CV curves at plethora of scan rates from 2 to 100 mV/s. Hence, Power law was adopted for further calculations which is described in Eqs. (8–9).

8 $$i= {i}_{cap}+{ i}_{diff}=a{v}^{b}$$

9 $$logi=loga+blogv$$

where i is the sum of diffusion controlled process (i_diff_) and surface capacitance controlled process (i_cap_), v represents scan rate, a and b are adjustable parameters where predominant charge storage mechanism was ascertained using b-values, as shown in Fig. S21 which depicts that all the samples completely reliance on ion diffusion. In order to further investigate the contribution of both the mechanism, the total capacity can be quantitively divided into two parts; k_1_v stands for capacitive effect and k_2_v^1/2^ for diffusion controlled effects asaccording to Eq. (10).

10 $$i(V)= {i}_{cap}+{ i}_{diff}= {k}_{1}v+ {k}_{2}{v}^{1/2}$$

**Fig. 10:**
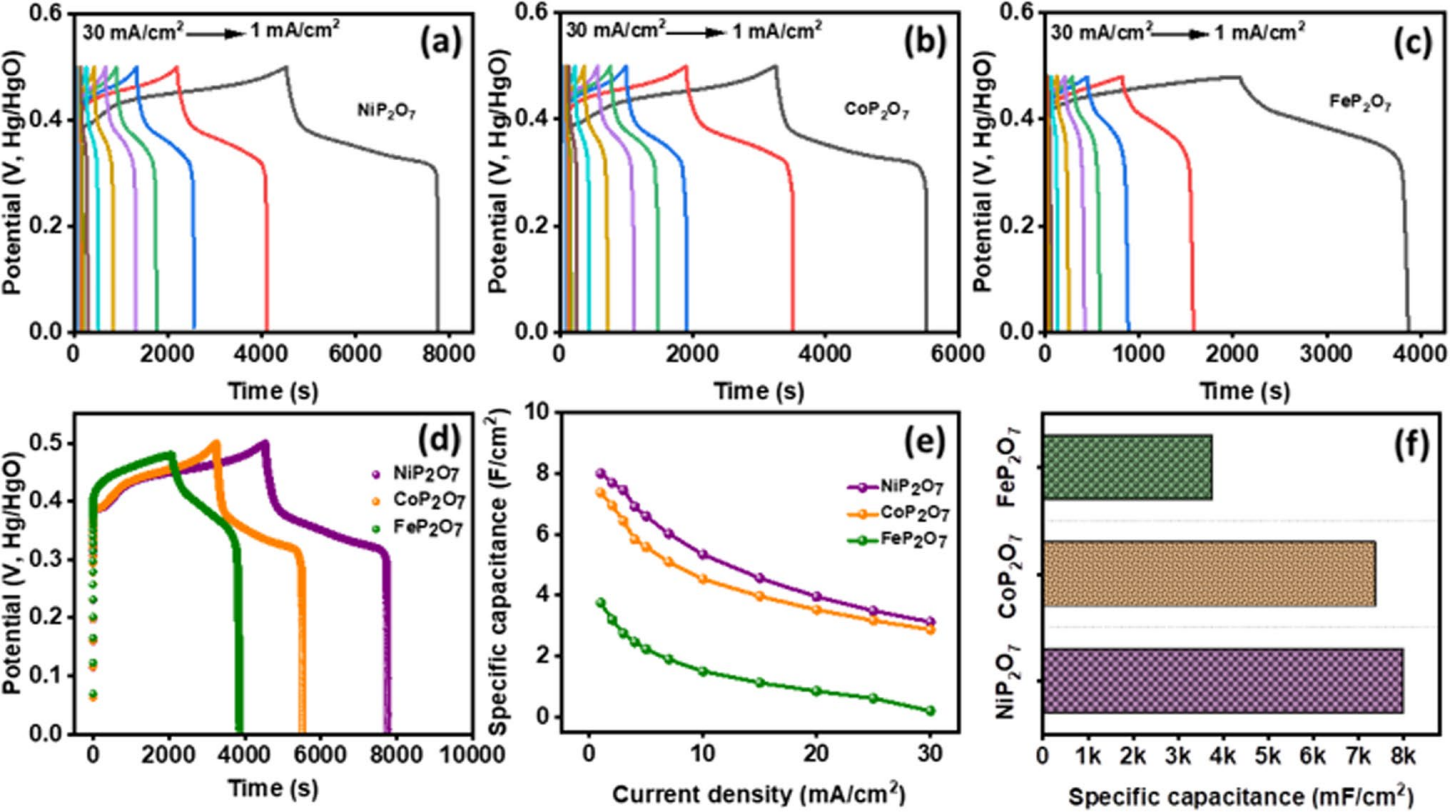
**a**–**c** Galvanostatic charging-discharging of the NiP_2_O_7_, CoP_2_O_7_, and FeP_2_O_7_ at different current densities ranging 30–1 mA/cm^2^, respectively, **d** Charge–discharge plot of NiP_2_O_7_, CoP_2_O_7_, and FeP_2_O_7_ at 1 mA/cm^2^, **e** Specific capacitance values at different current densities, and **f** Bar plot comparing specific capacitance of NiP_2_O_7_, CoP_2_O_7_, and FeP_2_O_7_ at 1 mA/cm^2^

The k_1_v and k_2_v^1/2^ contributions is illustrated for NiP_2_O_7_, CoP_2_O_7_, and FeP_2_O_7_ from 2 to 100 mV/s of scan rate in Figs. S22–S24. At low scan rate of 10 mV/s the k_1_v and k_2_v^1/2^ contributions were 5, 11, and 26% and 95, 89, and 74% for NiP_2_O_7_, CoP_2_O_7_, and FeP_2_O_7_, respectively, whereas, at 100 mV/s the contributions tuned to 13, 28, and 52% and 87, 72, and 48% for NiP_2_O_7_, CoP_2_O_7_, and FeP_2_O_7_, respectively. On the contrary of both the mechanisms, the diffusion dominated over capacitive nature in NiP_2_O_7_ with least involvement of surface capacitance. Therefore, it reflects that the NiP_2_O_7_ possess high rate of ingression of anions deep inside the nanomaterial than regression of the ions. Thus it takes a longer time to completely discharge of ions out of the material to the electrolyte. It is clearly visible that the diffusion was responsible to influence the charge barring property of the material. Furthermore, the charge holding ability of the electrodes were tested under the norms of galvanostatic charge–discharge (GCD) method and was performed at a different current densities of 1–30 mA/cm^2^ ran at a potential window of 0.0–0.5 V in a 3 M KOH. Discharge time is the indespensable parameter for the electrochemical performcance for supercapacitor and greater time indicates higher specific capacitance. The specific capacitance (C_s_) of the electrodes was derived by using the discharge profile of the GCD as shown in Fig. 10a–c, using Eq. (11) where i is current, Δt is discharge time drew from GCD plots, cm^2^ is the area of the electrodes, and Δv is the potential window. Herein, the GCD curve is disintegrated into three pillars; fast potential drop, plateau regime and sharp decay. The pioneer decay of potenial is due to internal resistance, intermediate regime of GCD dedicated to plateau due to faradic redox reaction where, NiP_2_O_7_ displayed a plateau region but FeP_2_O_7_ showed slant nature during discharge, indicating amorphosity of the material (surface capacitance). The GCD plot at higher current densities, such as 15, 20, 25 and 30 mA/cm^2^ showed a gradual decrease in the discharge time as a consequence of increased voltage drop and insufficient active electrode material took part in redox reactions at higher current densities. The comparitive plot of GCD for NiP_2_O_7_, CoP_2_O_7_, and FeP_2_O_7_ at a specific current density of 1 mA/cm^2^ was graphed in Fig. 10d to enumerates the discharge time taken and behavior of the curve. At last in the discharge process, the sudden drop of potenial was recorded which points the formation of double layer on the surface of the electrode.

11 $$Specific\;capacitance = I\Delta t/{\text{cm}}^{2} \Delta v$$

The calculated specific capacitance from the discharge curves is shown in Fig. 10e in the function of current densities. It was evident that the NiP_2_O_7_ outcasted better charge holding property with a maximum specific capacitance than other two samples over different range of current density. In Fig. 10f bar plot shows the comparison of specific capacitance at 1 mA/cm^2^, where greatest discharge time corresponds to NiP_2_O_7_ acquired highest specific capacitance of 7986 mF/cm^2^ (7.986 F/cm^2^) than the CoP_2_O_7_ (7363 mF/cm^2^) and FeP_2_O_7_ (3745 mF/cm^2^). Very astounding values of NiP_2_O_7_ is because of the agglomerated crystalline structured nanoparticles on the Ni-foam which facilitated more grain boundaries and higher surface area, further improving a more significant number of active sites for OH^−^ ions. The obtained values for specific capacitance of the three novel electrode materials are better than the already reported work in the past. For instance, nanosheets of Co_3_O_4_/CC (400 mF/cm^2^ @ 4 mA/cm^2^) [63], MnO@C composite (720 mF/cm^2^ @ 4 mA/cm^2^) [63], nanoneedle arrays of NiCo_2_O_4_ (0.99 F/cm^2^ @ 5.56 mA/cm^2^), [64] Na-doped Ni_2_P_2_O_7_//AC (22 mF/cm^2^ at 1 mA/cm^2^) [64], Ni_2_P_2_O_7_/Co_2_P_2_O_7_ nanograss array (2074 F/g @ 5 A/g) [37], Ni_3_(PO_4_)_2_/RGO/Co_3_(PO_4_)_2_ composite (1137.2 F/g at 0.5 A/g) [65], MnFe_2_O_4_/graphene/polyaniline (241 F/g @ 0.5 mA/cm^2^) [66], C_3_N_4_-1/Ni_2_P_2_O_7_ (4.4 F/cm^2^ @ 1 mA/cm^2^) [67], Li_2_Co_2_(MoO_4_)_3_ (1.03 F/cm^2^ @ 1 mA/cm^2^) [68], and Co_0.5_Ni_0.5_DHs/NiCo_2_O_4_/CFP (2.3 F/cm^2^ @ 2 mA/cm^2^) [69]. In that sense, the agglomerated spherical nanoparticles of the NiP_2_O_7_ as discussed in SEM investigation provided larger surface and volume ratio for ease in ions transportation in bulk.

**Fig. 11:**
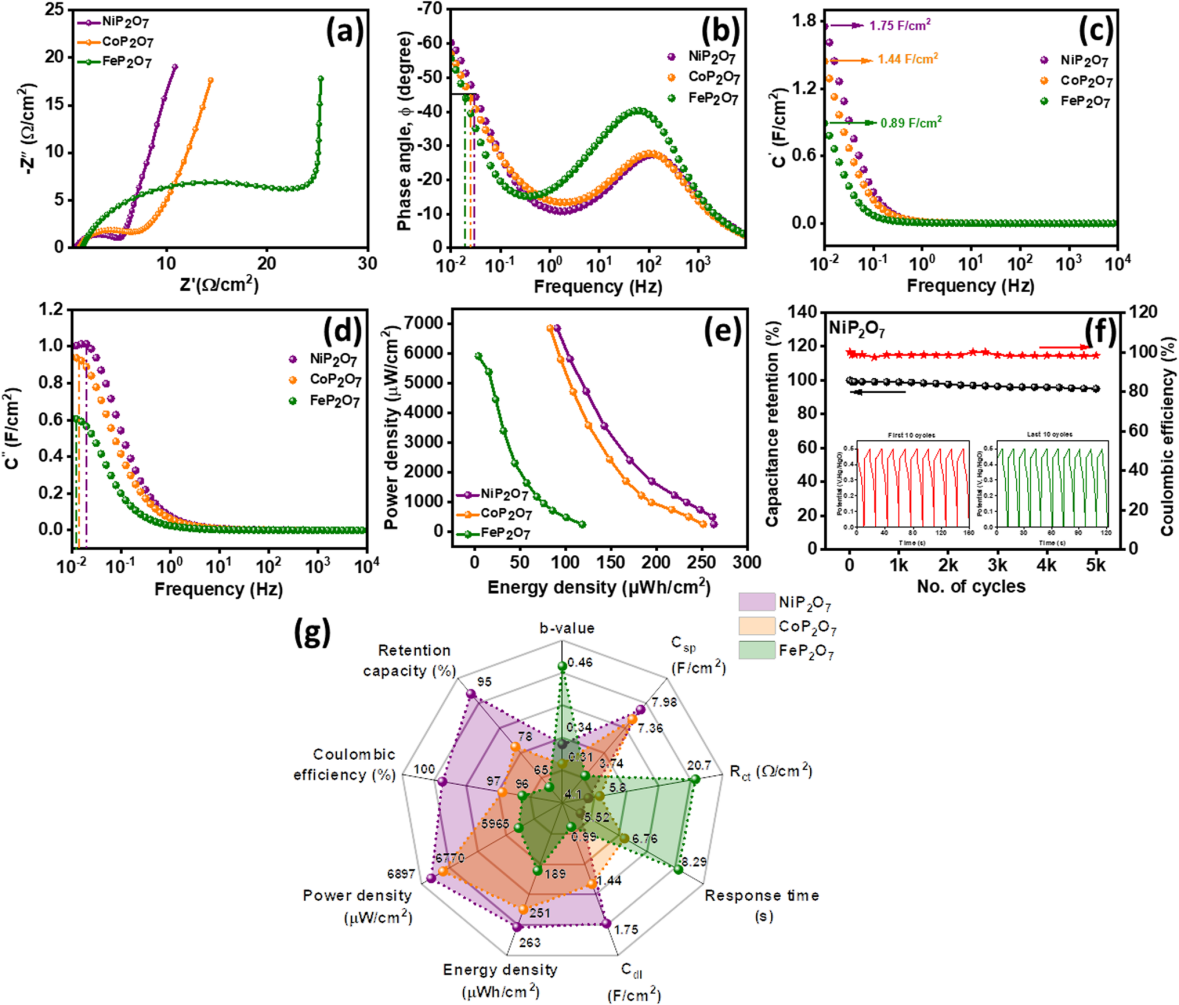
**a** EIS plot of NiP_2_O_7_, CoP_2_O_7_, and FeP_2_O_7_, **b** Bode phase angle for NiP_2_O_7_, CoP_2_O_7_, and FeP_2_O_7_ at 45°, **c** Real capacitance in function to frequency, **d** Imaginary capacitance in function to frequency, **e** Power density vs energy density of NiP_2_O_7_, CoP_2_O_7_, and FeP_2_O_7_, and **f** Stability plot of capacitance retention and coulombic efficiency for NiP_2_O_7_, **g** Radar plot comparing nine figures of merit: b-value, specific capacitance (C_sp_), charge transfer resistance (R_ct_), response time, double layer capacitance (C_dl_), energy density, power density, coulombic efficiency, and capacitance retention

To inculcate the further comprehensive understanding on the charge transfer route and electron transport during the electrochemical process. Therefore, the electrochemical impedance spectrum were measured at room temperature over a frequency range of 0.01–10 k Hz in a 10 mV AC amplitude under open circuit conditions. Figure 11a shows the Nyquist plot derived from the EIS data, where the arc in the high frequency region reflects the reaction occuring at the electrode surface, corresponding to electron transfer and controls the kinetics of the electrode interface. Hence charge transfer resistance can be calculated by measuring the radii of the arc; directly proportional to eachother [57, 58]. The charge transfer resistance of the NiP_2_O_7_, CoP_2_O_7_, and FeP_2_O_7_ is 4.05, 5.84, and 20.77 Ω/cm^2^. The associated Randles circuit [58] for the Nyquist plot of the as synthesized material was demonstrated in Fig. S25. On the basis of EIS observation the NiP_2_O_7_ is a better electrochemical active material and a defined slope at a lower frequency region also manifest the excellent ingression of ions in the electrode material, proposing more efficient charge transport thus, pointing good capacitive behavior.

Apart from this, Bode phase angle plot (Fig. 11b) was taken into consideration, which directs the capacitive and inductive nature of the material. If phase angle is closer to − 90°, the material obeys capacitive behavior and behave as an ideal capacitor. The phase angle lesser than − 90° at lower frequency represents pseudocapacitive nature [55]. Herein, all the synthesized material lie between − 50° to − 60°, confirming their pseudocapacitive trait. Moreover, the charge holding parameter and frequency correspondance to − 45° phase angle is defined as figure of merit for supercapacitor materials and response time was calculated and it was found that the NiP_2_O_7_ acquired the least response time of 5.521 s to transfer charges. However, the response time (Eq. 12) for CoP_2_O_7_ and FeP_2_O_7_ are 6.765 and 8.298 s, respectively. Hence, the less response time and charge transfer resistance of the NiP_2_O_7_ confirmed the improved kinetics.

The frequency dependent capacitance (C) is a addition of real ($C^{\prime}$) and imaginary capacitance (C″) (Eq. 13) [70]. Under that line, the double layer capacitance (C_dl_) was analyzed by plotting frequency dependent real capcitance ($C^{\prime}$) curve as shown in Fig. 11c, measured by using Eq. 14, where, Z″ and |Z| signifies the imaginary part (resistance), and modulus of total impedance, respectively and recorded that the capacitance value increases from zero to near saturation [71]. The C_dl_ value for NiP_2_O_7_ (1.75 F/cm^2^) is higher than CoP_2_O_7_, and FeP_2_O_7_, directing highest power density. Moreover, Fig. 11d elucidates imaginary capacitance to frequency (Eq. 15), where Z′ is the real part (resistance), which reassist the response time of NiP_2_O_7_, CoP_2_O_7_, and FeP_2_O_7_ is 7.722, 11.312, and 13.227 s and matches the trend of response time calculated by using phase angle plot, indicating NiP_2_O_7_ offered better energy density. Additionally, the relationship between power and energy densities were analyzed using the Ragone plot in Fig. 11e. With the niches in power density, the energy density depreciates. The maximum power density recorded for NiP_2_O_7_, CoP_2_O_7_, and FeP_2_O_7_ is 6.897, 6.870, and 5.965 mW/cm^2^ and energy density for the three materials are 0.263, 0.251, and 0.118 mWh/cm^2^, respectively. Thus NiP_2_O_7_ electrode commanding on power and energy density over other electrodes can be used for battery-supercapacitor hybrid device [72].

12 $$Response\;time = 1/2\pi f$$

13 $$\left( f \right) = C^{{\prime }} \left( f \right) + iC^{{\prime \prime }} \left( f \right),\quad {\text{where}}\;i = \sqrt { - 1}$$

14 $$C^{{\prime }} \left( f \right) = \frac{{ - Z^{{\prime \prime }} \left( f \right)}}{{2\pi f\left| {Z\left( f \right)} \right|^{2} }}$$

15 $$C^{{\prime \prime }} \left( f \right) = \frac{{ - Z^{{\prime }} \left( f \right)}}{{2\pi f\left| {Z\left( f \right)} \right|^{2} }}$$

Stability is considered as a crucial parameter for real life application. Thereby, the cycling stability was carried out for 5000 charge–discharge cycles, Fig. 11f, Figs. S26 and S27 shows the multiple charge–discharge cycles, capacitance retention and coulombic efficiency of the NiP_2_O_7_, CoP_2_O_7_, and FeP_2_O_7_, respectively, where NiP_2_O_7_ holds 97% of capacitance retention and 100% coulombic efficiency.

The Radar plot, as displays in Fig. 11g, compares 9 figure of merit, that is C_sp_, b-value, R_ct_, response time calculated using phase angle plot, C_dl_, energy desnity, power density, capacitance retention, and coulombic efficiency of NiP_2_O_7_, CoP_2_O_7_, and FeP_2_O_7_. The salient features depicted from the Radar plot and observations as follows; (1) The XRD reveals the high crystallinity of NiP_2_O_7_ than other samples, (2) EDX showed more percentage composition of Ni in NiP_2_O_7_ than other transition metals in CoP_2_O_7_ and FeP_2_O_7_, exhibiting more conversion of oxidation states in NiP_2_O_7_ and assist passivity to the ions feassibly, (3) thus numerous active sites are provided by NiP_2_O_7_ and better diffusion of ions as an effect of less R_ct_ and response time during electrochemical reaction, (4) highest value of C_dl_, C_sp_, power density, energy density and stability cordially contributing to reason that NiP_2_O_7_ showed improved and best candidate for supercapacitor application among other pyrophosphates.

## Conclusion

In summary, three composites, NiP_2_O_7_, CoP_2_O_7_, and FeP_2_O_7_ nanoparticles grown on Ni-foam, were prepared using hydrothermal strategy, and characterized via multiple analytical techniques. The prepared composites were tested for electrochemical water splitting and supercapacitors. The results indicated the superior performance of FeP_2_O_7_ composite towards OER and HER, displaying the lowest overpotential of 220 and 241 mV at 10 mA/cm^2^, respectively. The DFT calculations revealed that FeP_2_O_7_ had a higher distribution of DOS over the fermi level than CoP_2_O_7_ and NiP_2_O_7_, which improved its electronic characteristics and contributed to its superior electrochemical performances towards OER, and HER. Moreover, NiP_2_O_7_, with its higher percentage composition of Ni on the Ni-foam, which permits more Ni to convert into its oxidation states and come back to its original oxidation state during supercapacitor testing, was found to have the highest specific capacitance and remarkable cycle stability due to its high crystallinity. This work can serve as an approach towards understanding of electronic alterations in transition metals via pyrophosphate species to design efficient materials for developing energy conversion and storage.

### Supplementary Information

**Additional file 1.**

## Data Availability

The datasets generated during and/or analyzed during the current study are available from the corresponding author on reasonable request.
