# Supplementary material for: Optimum iron-pyrophosphate electronic coupling to improve electrochemical water splitting and charge storage
Source: Discov Nano. 2023 Dec 4;18(1):148. doi: 10.1186/s11671-023-03937-y (PMC10695914; doi:10.1186/s11671-023-03937-y)
Supplement: Supplementary file 1 — Additional file 1. [file 11671_2023_3937_MOESM1_ESM.docx]

**Optimum Iron-Pyrophosphate Electronic Coupling to Improve Electrochemical Water Splitting and Charge Storage**

**Rishabh Srivastava^1,2^, Himanshu Chaudhary^2^, Anuj Kumar^3*^, Felipe M. de Souza^2^, Sanjay R. Mishra^4^, Felio Perez^5^, and Ram K. Gupta^2,6*^**

^1^ Department of Physics, Pittsburg State University, Pittsburg, KS 66762, USA

^2^ National Institute for Materials Advancement, Pittsburg State University, Pittsburg, KS 66762, USA

^3^ Nano-Technology Research Laboratory, Department of Chemistry, GLA University, Mathura, Uttar Pradesh 281406, India

^4^Department of Physics and Materials Science, The University of Memphis, Memphis, TN 38152, USA

^5^Integrated Microscopy Center, The University of Memphis, Memphis, TN 38152, USA

^6^Department of Chemistry, Pittsburg State University, Pittsburg, KS 66762, USA

*Corresponding authors: Anuj Kumar ([anuj.kumar@gla.ac.in](mailto:anuj.kumar@gla.ac.in)), Ram K. Gupta (ramguptamsu@gmail.com)


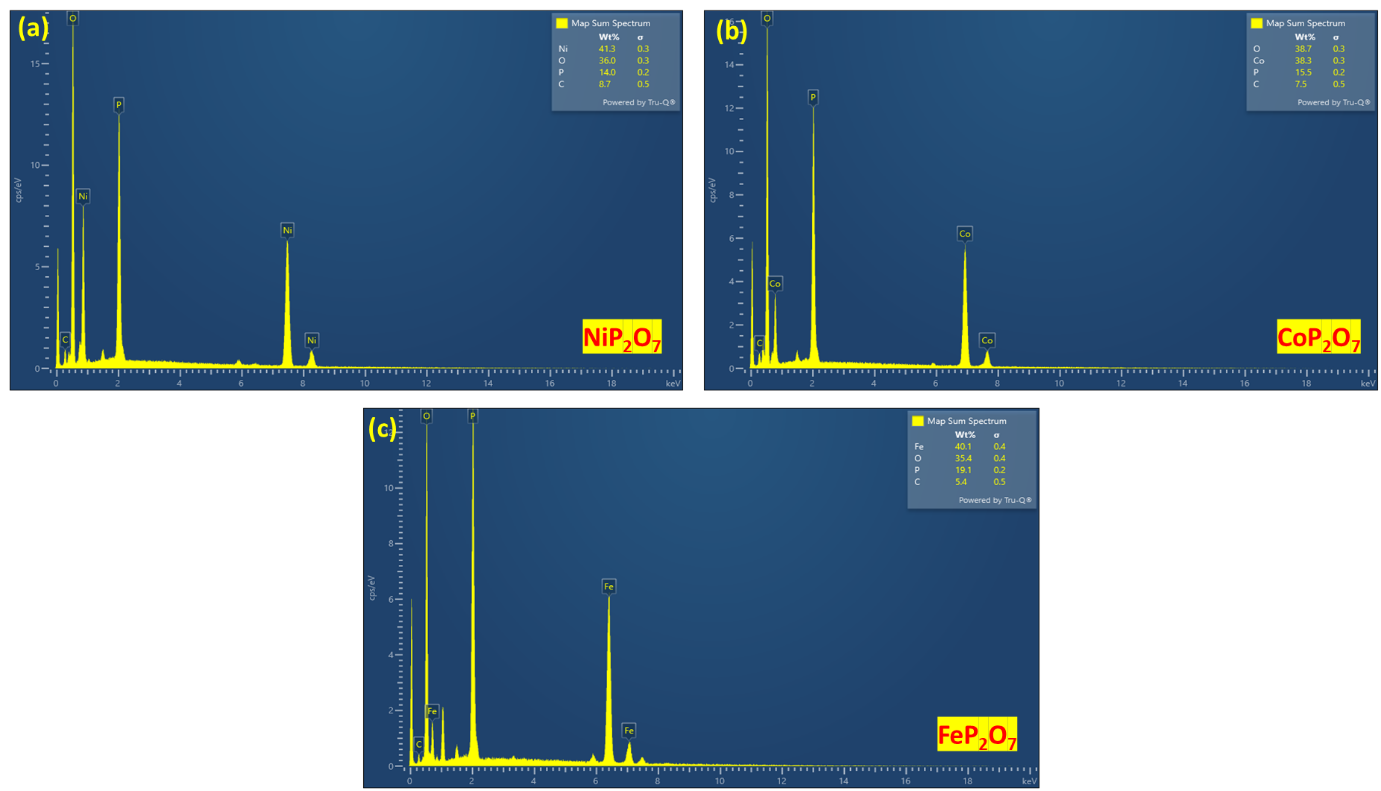


**S1:** EDX analysis (20 kV) of (a) Ni Kal, P Kal, and O Kal emission lines for NiP_2_O_7_, (b) Co Kal, P Kal, and O Kal emission lines for CoP_2_O_7_, and (c) Fe Kal, P Kal, and O Kal emission lines for FeP_2_O_7_ at 10μm.


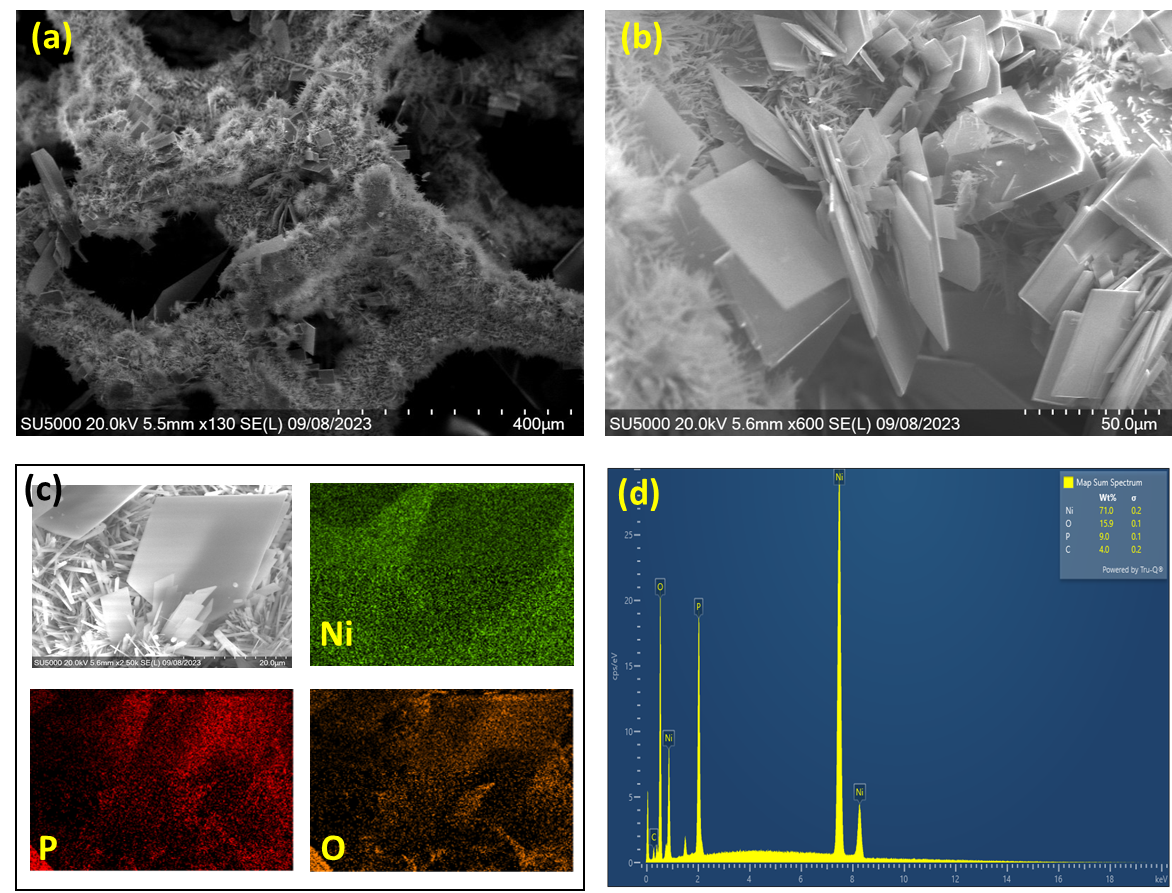


**S2:** SEM images of grown NiP_2_O_7_ on Ni-foam before testing (a) at 400 μm, (b) at 50 μm, (c) enlarged SEM image of NiP_2_O_7_ and elemental mapping (20 kV) of Ni Kal, P Kal, and O kal, and (d) EDX analysis of Ni Kal, P Kal, and O kal emission lines for NiP_2_O_7_.


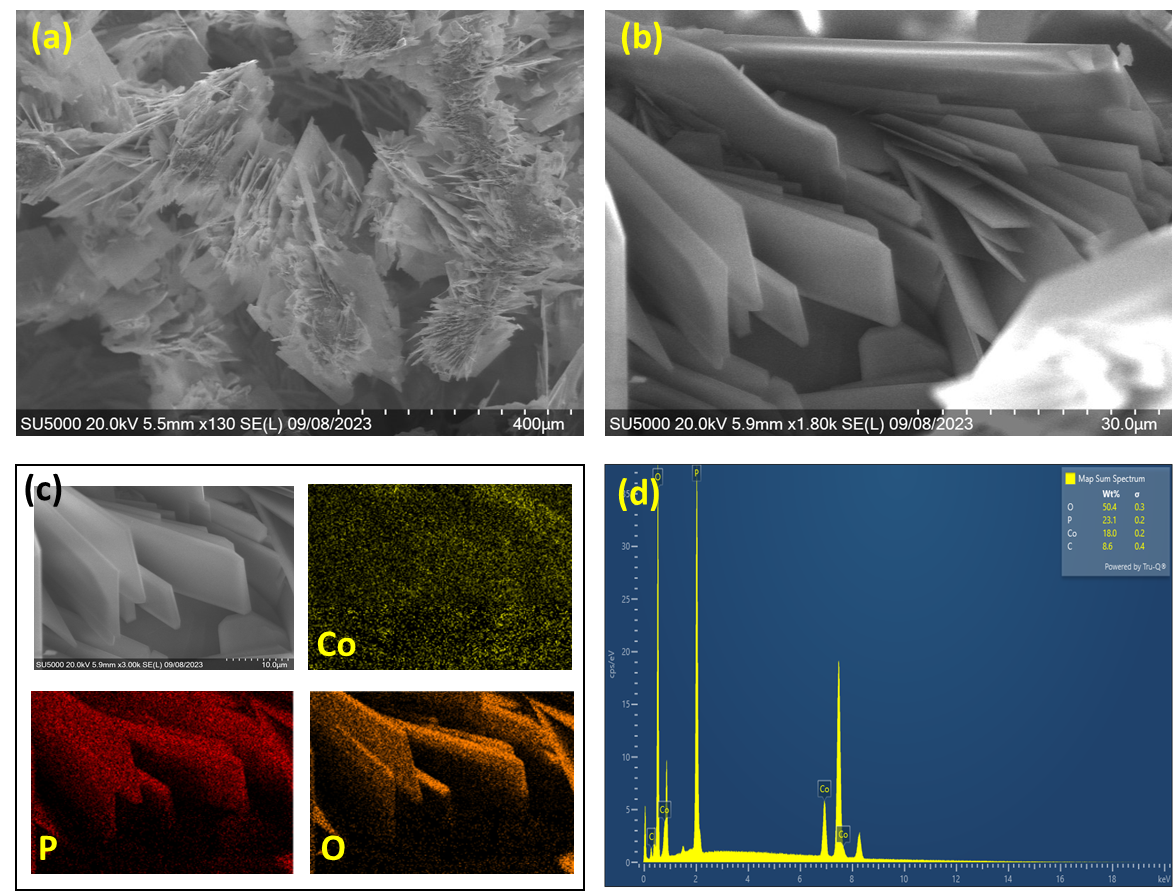


**S3:** SEM images of grown CoP_2_O_7_ on Ni-foam before testing (a) at 400 μm, (b) at 30 μm, (c) enlarged SEM image of CoP_2_O_7_ and elemental mapping (20 kV) of Co Kal, P Kal, and O kal, and (d) EDX analysis of Co Kal, P Kal, and O kal emission lines for CoP_2_O_7_.


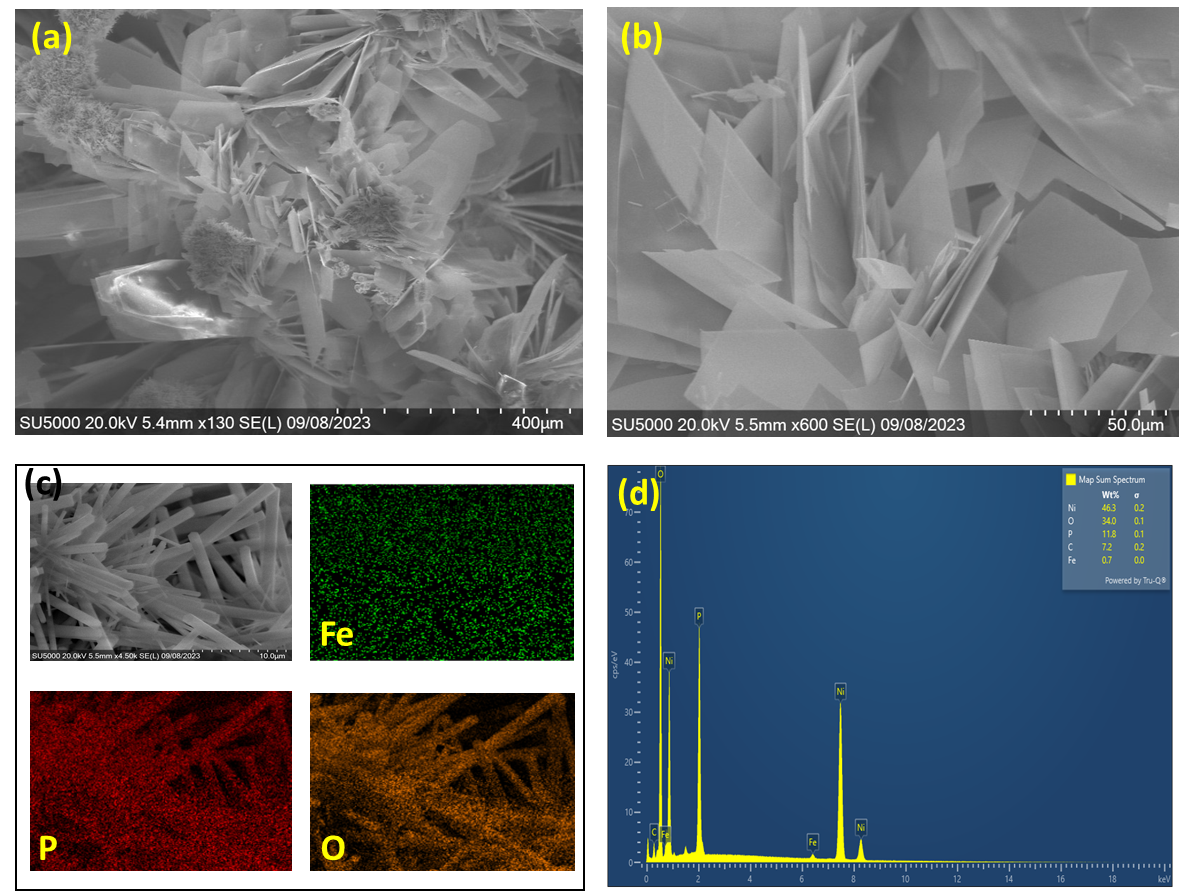


**S4:** SEM images of grown FeP_2_O_7_ on Ni-foam before testing (a) at 400 μm, (b) at 50 μm, (c) enlarged SEM image of FeP_2_O_7_ and elemental mapping (20 kV) of Fe Kal, P Kal, and O kal, and (d) EDX analysis of Fe Kal, P Kal, and O kal emission lines for FeP_2_O_7_.

**S5:** High-resolution spectrum of P 2p for CoP_2_O_7_

**S6:** High-resolution spectrum of O 1s for CoP_2_O_7_

**S7:** High-resolution spectrum of Fe 2p for FeP_2_O_7_

**S8:** High-resolution spectrum of P 2p for FeP_2_O_7_

**S9:** High-resolution spectrum of O 1s for FeP_2_O_7_

**S10:** Comparision of HER overpotential among all the prepared electrodes at 5, 10, and 50 mA/cm^2^

**S11:** LSV curves after and before 1000 cycles of cyclic voltammetry for NiP_2_O_7_ sample

**S12:** LSV curves after and before 1000 cycles of cyclic voltammetry for CoP_2_O_7_ sample

**S13:** Comparision of OER overpotential among all the prepared electrodes at 5, 10, and 50 mA/cm^2^.

**S14:** OER Polarization curve over 1k cycles for NiP_2_O_7_

**S15:** OER Polarization curve over 1k cycle of CoP_2_O_7_.

**S16:** Electrochemical Impedance spectroscopy of NiP_2_O_7_

**S17:** Electrochemical Impedance spectroscopy of CoP_2_O_7_


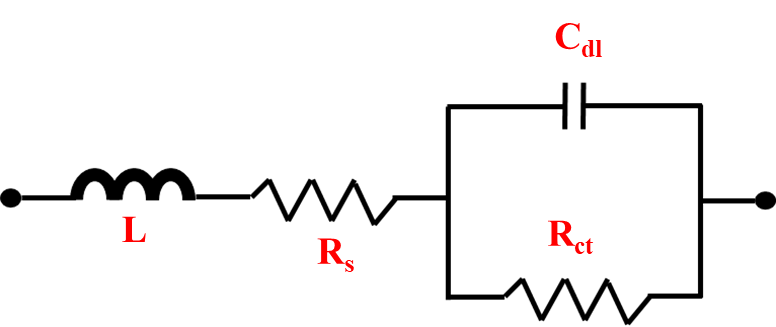


**S18:** Randle circuit according to Nyquist plot obtained for NiP_2_O_7_, CoP_2_O_7_, and FeP_2_O­_7_.

**Table S1:** Rct (Ω/cm^2^) comparision of NiP2O7, CoP2O7, and FeP2O7 at 0.45, 0.5, 0.55, and 0.6 V

| Sample Name | **@0.45 V** | **@0.5 V** | **@0.55 V** | **@0.6 V** |
| --- | --- | --- | --- | --- |
| NiP_2_O_7_ | 13.27 Ω/cm^2^ | 11.02 Ω/cm^2^ | 5.14 Ω/cm^2^ | 3.30 Ω/cm^2^ |
| CoP_2_O_7_ | 42.19 Ω/cm^2^ | 36.45 Ω/cm^2^ | 30.01 Ω/cm^2^ | 25.21 Ω/cm^2^ |
| FeP_2_O_7_ | 4.89 Ω/cm^2^ | 4.71 Ω/cm^2^ | 4.58 Ω/cm^2^ | 3.76 Ω/cm^2^ |


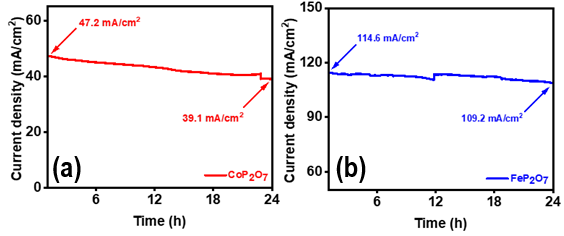


**S19:** Chronoamperometry curve of (a) CoP_2_O_7_, and (b) FeP_2_O_7_ over 24 h.


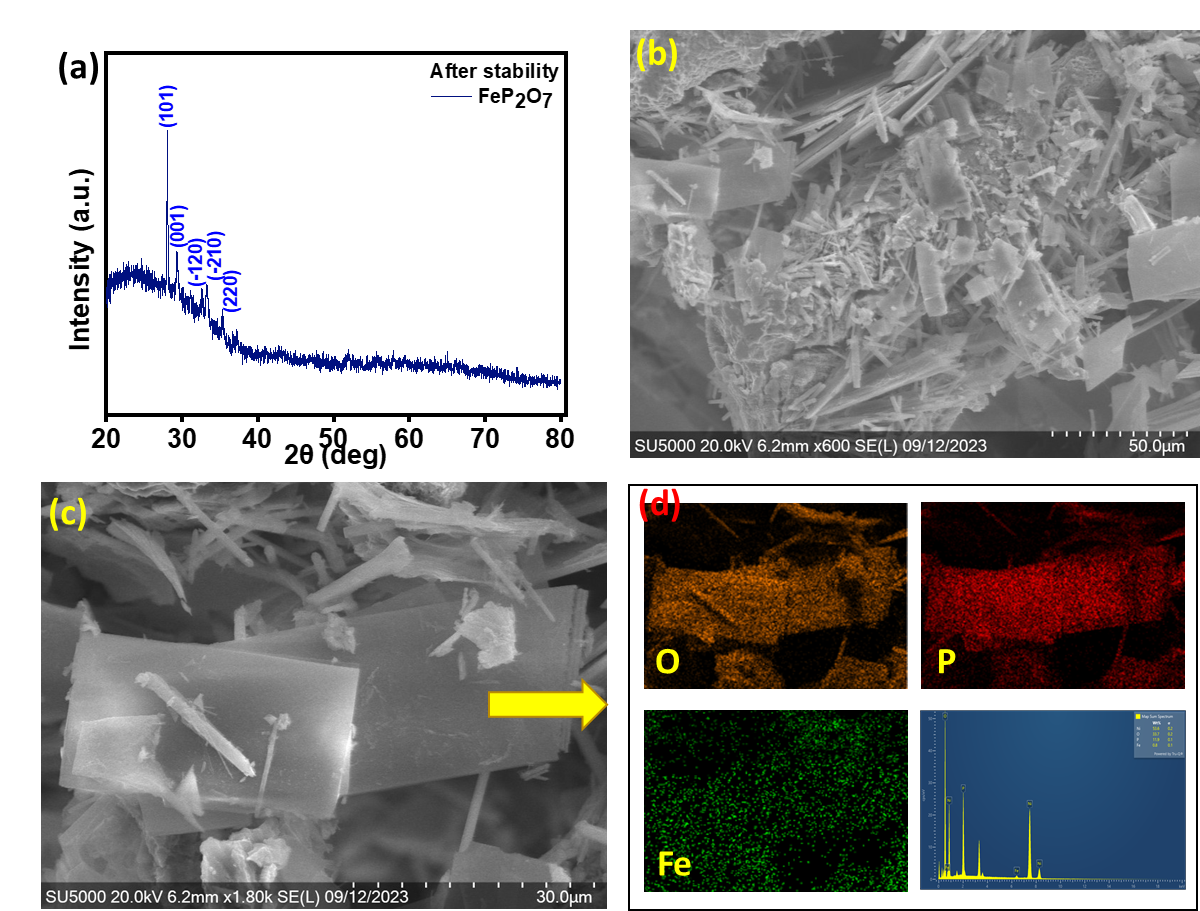


**S20:** (a) XRD of FeP_2_O_7_ after overall water-splitting stability test, SEM images of FeP_2_O_7_ electrode after overall water-splitting (b) at magnification of 50 μm, and (c) 30 μm, (d) elemental mapping (20 kV) of O Kal, P Kal, and Fe kal, and EDX analysis of Fe Kal, P Kal, and O kal emission lines for FeP_2_O_7_ at 30 μm of magnification.

**S21:** Power-law dependence of anodic currents at various scan rates.

**S22:** diffusion and capacitive controlled contribution at various scan rates ranging from 2-100 mV/s for NiP_2_O_7_.

**S23:** diffusion and capacitive controlled contribution at various scan rates ranging from 2-100 mV/s for CoP_2_O_7_.

**S24:** diffusion and capacitive controlled contribution at various scan rates ranging from 2-100 mV/s for FeP_2_O_7_.

**
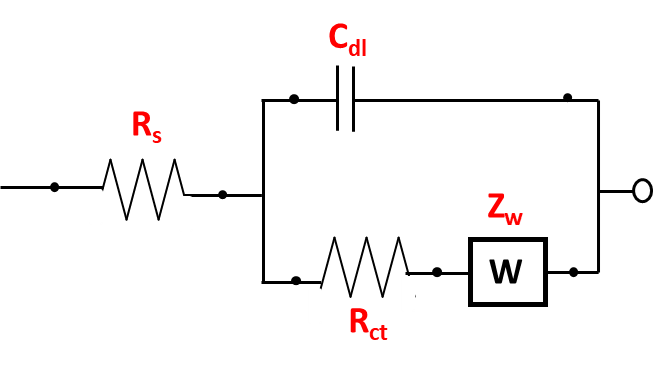
**

**S25:** Randle circuit according to Nyquist plot obtained for NiP_2_O_7_, CoP_2_O_7_, and FeP_2_O­_7_.

**S26:** Capacitance retention and coulombic efficiency plot of CoP_2_O_7_.

**S27:** Capacitance retention and coulombic efficiency plot of FeP_2_O_7_.
